# A Robust Actin Filaments Image Analysis Framework

**DOI:** 10.1371/journal.pcbi.1005063

**Published:** 2016-08-23

**Authors:** Mitchel Alioscha-Perez, Carine Benadiba, Katty Goossens, Sandor Kasas, Giovanni Dietler, Ronnie Willaert, Hichem Sahli

**Affiliations:** 1 Electronics and Informatics Dept (ETRO), AVSP Lab, Vrije Universiteit Brussel, Brussels, Belgium; 2 VUB-EPFL International Joint Research Group (IJRG) NanoBiotechnology and NanoMedicine (NANO), Brussels, Belgium; 3 Laboratoire de Physique de la Matière Vivante (LPMV), EPFL, Cubotron, Lausanne, Switzerland; 4 Department of Bioengineering Sciences (DBIT), Vrije Universiteit Brussel, Brussels, Belgium; 5 Interuniversity Microelectronics Centre (IMEC), Heverlee, Belgium; Burnham Institute for Medical Research, UNITED STATES

## Abstract

The cytoskeleton is a highly dynamical protein network that plays a central role in numerous cellular physiological processes, and is traditionally divided into three components according to its chemical composition, i.e. actin, tubulin and intermediate filament cytoskeletons. Understanding the cytoskeleton dynamics is of prime importance to unveil mechanisms involved in cell adaptation to any stress type. Fluorescence imaging of cytoskeleton structures allows analyzing the impact of mechanical stimulation in the cytoskeleton, but it also imposes additional challenges in the image processing stage, such as the presence of imaging-related artifacts and heavy blurring introduced by (high-throughput) automated scans. However, although there exists a considerable number of image-based analytical tools to address the image processing and analysis, most of them are unfit to cope with the aforementioned challenges. Filamentous structures in images can be considered as a piecewise composition of quasi-straight segments (at least in some finer or coarser scale). Based on this observation, we propose a three-steps actin filaments extraction methodology: (i) first the input image is decomposed into a ‘cartoon’ part corresponding to the filament structures in the image, and a noise/texture part, (ii) on the ‘cartoon’ image, we apply a multi-scale line detector coupled with a (iii) quasi-straight filaments merging algorithm for fiber extraction. The proposed robust actin filaments image analysis framework allows extracting individual filaments in the presence of noise, artifacts and heavy blurring. Moreover, it provides numerous parameters such as filaments orientation, position and length, useful for further analysis. Cell image decomposition is relatively under-exploited in biological images processing, and our study shows the benefits it provides when addressing such tasks. Experimental validation was conducted using publicly available datasets, and in osteoblasts grown in two different conditions: static (control) and fluid shear stress. The proposed methodology exhibited higher sensitivity values and similar accuracy compared to state-of-the-art methods.

## Author Summary

We propose a novel actin filaments cytoskeleton analysis framework that allows extracting quasi-straight individual fibers in a robust manner, and provides their respective *position*, *orientation*, and *length* as output. The proposed framework is defined as a three-steps processing sequence, that can explicitly cope with high-throughput imaging related issues, such as noise/artifacts presence and heavy blurring, and can similarly process artifacts-free and well-focused images.

This is a PLoS Computational Biology Methods paper.

## Introduction

The actin cytoskeleton plays a fundamental role in numerous cellular processes such as cell growth [1, 2], proliferation and migration [3–5], differentiation [6–9] and apoptosis [10]. It is a highly dynamical structure that polymerizes and depolymerizes in a timeframe of minutes according to different intra- or extra-cellular stimuli. It is composed by a set of actin filaments organized in a complex three-dimensional network spanning within the cell, and is anchored to the extra-cellular matrix via trans-membrane proteins (integrins) and focal-adhesion related proteins (i.e. paxilin, zyxin, vinculin, and others). Such proteins mediate the cells mechanosensing of the microenvironment, allowing the cytoskeleton to reactively adapt to external mechano-stimuli [11].

Mechanical stimulation can cause significant variations in the cells geometry, triggering actin filaments polymerization/depolymerization to balance the applied extra-cellular forces. The actin polymerization response produces filaments, which in (typically 10 ∼ 30) bundles form the actin stress fibers (AF) [12]. They are central in order to study mechanosensing and mechanotransduction related pathways, and unveil the underlying mechanisms that regulate many of the aforementioned cellular processes [12].

**Fig 1:**
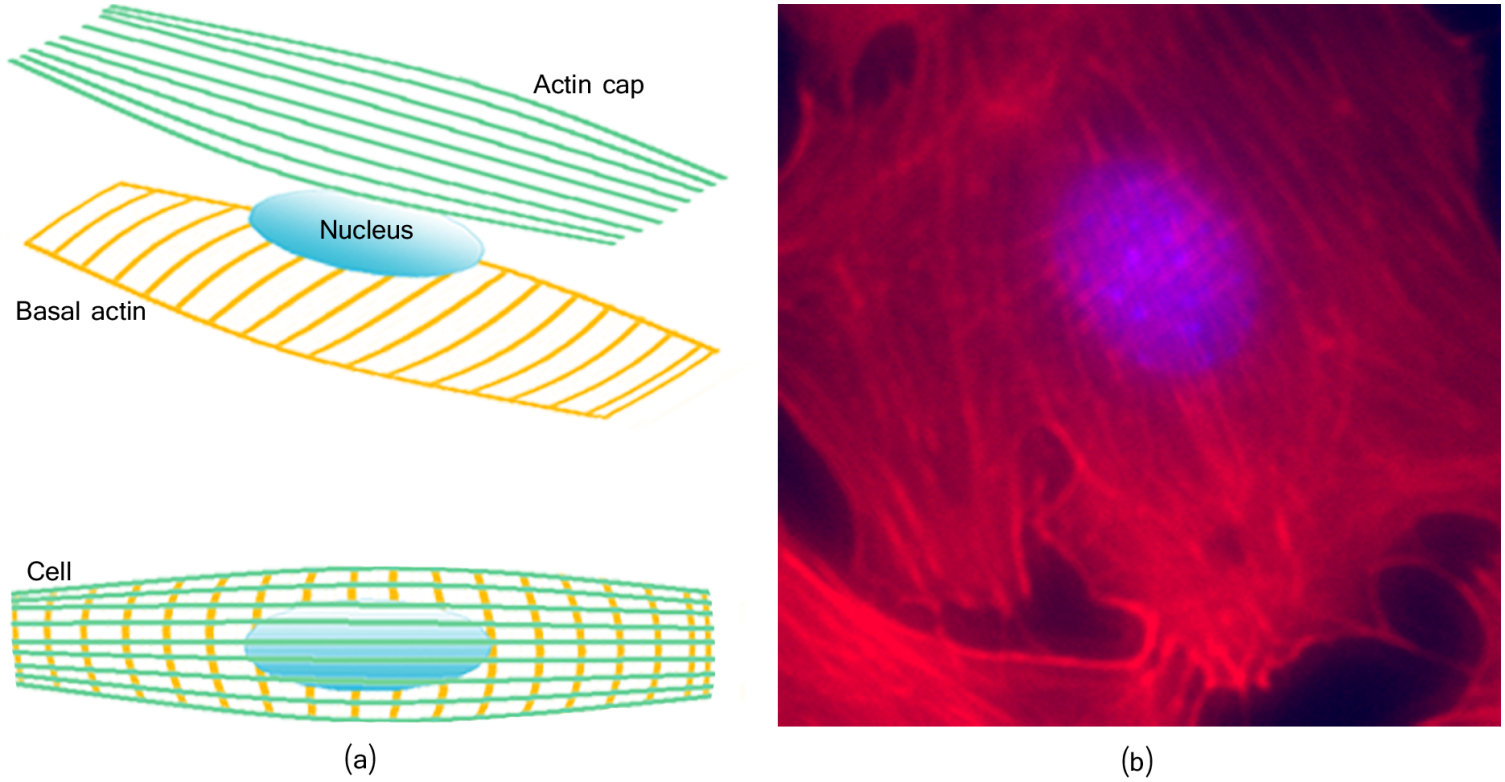
Obtained cytoskeleton image can include some degrees of blurring. (a) Illustration of the cap and basal actin layers. (b) Image of a rat osteoblast (bone producing cell) with phalloidin (red) stained actin cytoskeleton and dapi stained nucleus (blue).

Several stress inducing protocols are well established to approach cytoskeleton behaviors, including fluid shear stress [13, 14], (simulated) micro-gravity [15, 16], cyclic stretch [11, 17, 18], among others. Cellular dynamics under such stress conditions are then studied by classical fluorescence or confocal microscopy, on fixed or alive cells. The extracted actin stress fibers provide the necessary information for further analysis. However, although there is a considerable number of image-based analytical tools [19–21] for the image processing stage, most of them are unfit to cope with blurring and imaging-related artifacts introduced by (high-throughput) automated scans or the imaging of certain filaments. This is the case of (2D) cytoskeleton imaging where actin cap and basal actin filaments are located in different (apical and basal) focal planes (see Fig 1), causing the fibers image to exhibit at least some degree of blurring. Similarly, automated scans often produce blurred images including noise and artifacts.

Even in such cases, one can assume that actin fibers are a piecewise composition of quasi-straight segments (at least in some finer or coarser scale), and the directionality of those segments can serve as guide to track individual (blurred and potentially overlapping) filaments. On the contrary, noise and artifacts present in the image are unlikely to exhibit the fibers specific geometry. Thus, quasi-straight clues present in the image can be associated to fibers, since such structures are unlikely to emerge from a random process (Helmholtz principle [22]).

To separate noise/texture from actin fibers, we propose using image decomposition [23–25]. Given an input image *f*, such approach decompose it as *f* = *u* + *v*, where the image *u* represents the ‘cartoon’ information, and *v* the ‘texture’ and/or noise image. Among the existing techniques, the authors of [23] proposed a morphological component analysis (MCA) based image separation which constructs a sparse representation of an image and separates the image into morphological components (MCs). This method assumes the ‘cartoon’ and ‘texture’ be represented via some known basis functions. We borrow the image decomposition approach of [23] and propose the curvelets model [26] as basis-function for the quasi-linear fiber content, and a wavelet model for the artifacts (texture/noise) content. Our motivation of using the curvelets transform is due to the fact that common denominators among actin filaments structures (as shown in Fig 1b) are, (i) they display anisotropic line-like features, (ii) they basically show behaviors of *C*^2^-continuous curves, and (iii) they are relatively smooth in the direction along the filament. Curvelets enables the possibility to directionally analyze an image with different angular resolutions in a single and effective transform [27]. Curvelets take the form of basis elements, which have elongated effective support; i.e., length > width [28]. This method is a good candidate for the detection of anisotropic structures of different lengths [27], such as actin filaments.

In this work, we propose a novel actin filaments cytoskeleton analysis framework that allows extracting quasi-straight individual fibers in a robust manner, and provides their respective *position*, *orientation*, and *length* as output. The proposed framework can explicitly cope with high-throughput imaging related issues, such as noise/artifacts presence and heavy blurring, and can similarly process artifacts-free and well-focused images. During experiments, the proposed model was able to extract a higher number of individual fibers compared to other state-of-the-art models in several cytoskeleton images.

### State of the Art

Computer vision and image-analytical tools are essential in order to study the biology of cells [29]. Several approaches exist for the analysis of filamentous structures [19–21, 30–36], consisting in one or several sequential processing steps: pre-processing, filaments network segmentation, and individual fibers extraction.

**Fig 2:**
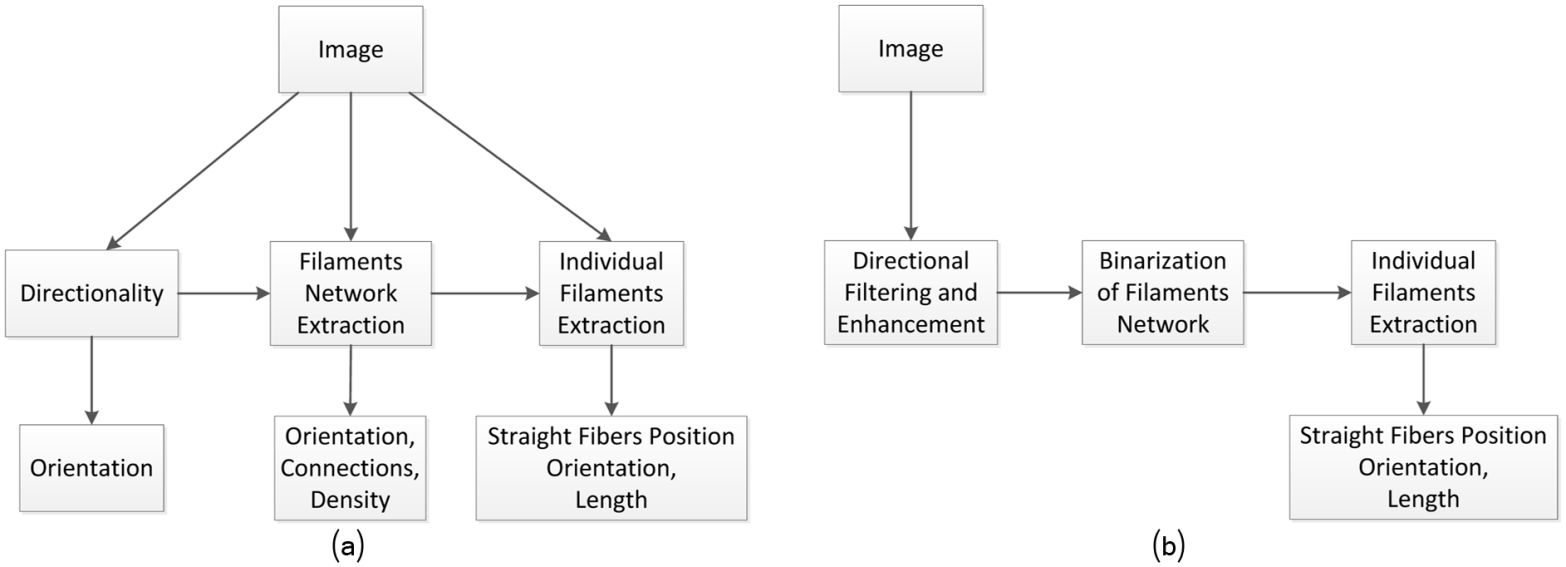
Different analysis strategies will determine the amount of information to be extracted from the image. (a) Possible fibers analysis strategies. (b) The strategy followed involve three sequential stages: directional filaments filtering and enhancement, filaments network segmentation and individual fibers extraction.

The filaments analysis problem can be solved in many different ways, as illustrated in Fig 2a. These solutions can be roughly organized in at least three main categories (middle layers in Fig 2a and 2b): based on filaments directionality, based on filaments network, and based on single-filaments extraction. The processing path (from the image to output) will naturally determine the computational burden, accuracy and the amount of information to be extracted from the image. The strategy we followed is depicted in Fig 2b.

In the first category, the filaments information is directly extracted from the pre-processed image [30, 35], sidestepping the foreground/background segmentation step. Segmentation-related errors were avoided at the cost of limiting the model applicability to directionality-related analysis [37] only. Networks-related approaches extract the filaments networks [19] after filtering, allowing to complement orientation information with motion and filaments density analysis [38]. One category of methods for the identification of fiber networks uses template matching [39], where prior knowledge about the target is incorporated into 3D (or 2D) templates. These template-based detection methods are more selective and impose a similarity function. In [40], actin filaments in cryo-ET data sets have been segmented using a stochastic template-based search, which combines a genetic algorithm and a bidirectional expansion strategy. Template matching methods does not resolve the possible template overlap when tracing converging filament branches, thus network junctions could be left undetected. Such problem has been solved in [41] where an active contour based method allowed increasing the segmentation robustness by incorporating prior information about the filament shape. Such approach explicitly models the linear nature of filaments.

The overall strategy of the network-based approaches is to extract the whole curvilinear network at once. In contrast, our work aims at providing information at the single-fiber level, where individual filament segments [20, 21, 32] are extracted. This allows to perform further analysis [11, 16, 42–46] taking into account fibers position, orientation, and length. However, errors introduced during the different processing stages can accumulate and, unless appropriate computational steps are taken, later analysis (statistical and other) can be compromised.

#### Directional filtering and edges enhancement

Decomposing an image into meaningful components is an important and challenging inverse problem in computer vision. Image filtering/denoising is an example of image decomposition, which separates the image into signal parts and noise parts. Laplace filters [47], steerable (directional) Gaussian [48], or a combination of them [21] have been used in the literature. More sophisticated techniques, such as Coherence-enhancement filters [49] are well designed to enhance flow-like structures and merge those with (small) discontinuities in a proper way, but they are unable to tackle blurring satisfactorily. In [44], the Radon transform has been used in the context of filaments detection, as a pre-processing step applied to the input image.

Under the perspective that we are addressing a content-specific computer vision application, i.e. separating filamentous structures from other image content, in this work we consider image decomposition as the process of separating an image in conceptually and theoretically different components [23–25]. In these methods a typical assumption is made that the given image is a linear mixture of several source images of more coherent origin. For instance, a nature image is linearly mixed by three coherent components, ‘cartoon’ structure image, ‘texture’ image and ‘noise’ image. The different components require content-specific modelling and representations, and motivate an analysis in two parallel channels. The structure part for 1D feature detection (edges, ridges, etc.), segmentation, object recognition and shape analysis, while the texture component can be used for surface analysis, etc. In the proposed framework, we first perform a sparse multi-source separation strategy [23, 50, 51] to separate the image content into two main parts (sources): the artifacts related content (considered as texture and/or noise) and the filaments related content. Edge enhancement and filament extraction is then applied on the image containing the filaments. We make use of a curvelets model [27] for the quasi-linear fiber content representation, and a wavelet model for the artifacts content. As indicated above, curvelets are ideal candidates for multi-scale edges and directional representations compared to other models.

#### Network binarization

The network segmentation requires separating those pixels belonging to the network from the rest, in a process known as binarization. Standard binarization techniques includes global thresholding, such as Otsu’s method [52], and local adaptive thresholding based neighborhood means; a combination of both is often used [21, 53]. Instead, taking into account the multi-scale directional nature of the fibers, we performed a multi-scale line segmentation similar to [34] but combined with adaptive local thresholding. The results provided the actin filament network but not yet the individual fibers.

#### Fibers extraction

Individual filaments extraction is based on line segment detectors. Hough-based detection approaches exhibit a high computational burden, while light models [32] can miss line segments whose probability is near the decision boundary. Extraction strategies such as the one proposed in [21] are efficient, but they fail to detect several heavy-blurred filaments. We propose a segments extraction algorithm that detects overlapping segments based on a multi-scale directional line response. Quasi-straight line segments of fixed length are extracted at some specific scale, and iteratively merged in order to obtain the final individual fibers.

## Materials and Methods

**Fig 3:**
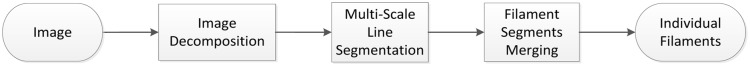
The proposed framework is based on a specific three stage sequence of processing steps.

In this work, we propose a sequential three stage processing framework as illustrated in Fig 3. It involves a multi-source filaments separation for the first stage, a multi-scale line detection for the second stage, and a filaments segments merging algorithm for the last stage. The output of each processing step serve as input to the next step, detailed in the following sections.

### Cells Culture

MC3T3-E1 cells, established as an osteoblastic cell line, were provided from Sigma (99072810). Osteoblasts were grown in alpha-modified minimal essential medium (*α*-MEM; Life Technologies) supplemented with 10% fetal bovine serum (Life Technologies), 1% L-glutamin (Life Technologies) and 1% penicillin/streptomycin (Life Technologies) in an incubator at 5% CO_2_ heated at 37°C. The medium was changed twice weekly, and the cells were subcultured into 75 cm^2^ culture flasks by detaching them gently after a brief PBS rinsing step followed by Trypsin treatment once the cells were reaching subconfluency. For mechanical stimulation, MC3T3-E1 cells were plated into Ibidi *μ*-slide device (*μ*-Slide I 0.4 Luer, ibiTreat from Ibidi) at a concentration of 1.5 × 10^4^ cells/mL. After overnight culture, the medium was replaced by *α*-MEM (powder exempt of Bicarbonate, Life Technologies) 10% FBS 1% L-glutamin 1% penicillin/streptomycin 25 mM HEPES (Life Technologies) adjusted to pH 7.4 for 4 hours in the incubator without CO_2_ at 37°C.

### Fluid Shear Stress Induction

**Fig 4:**
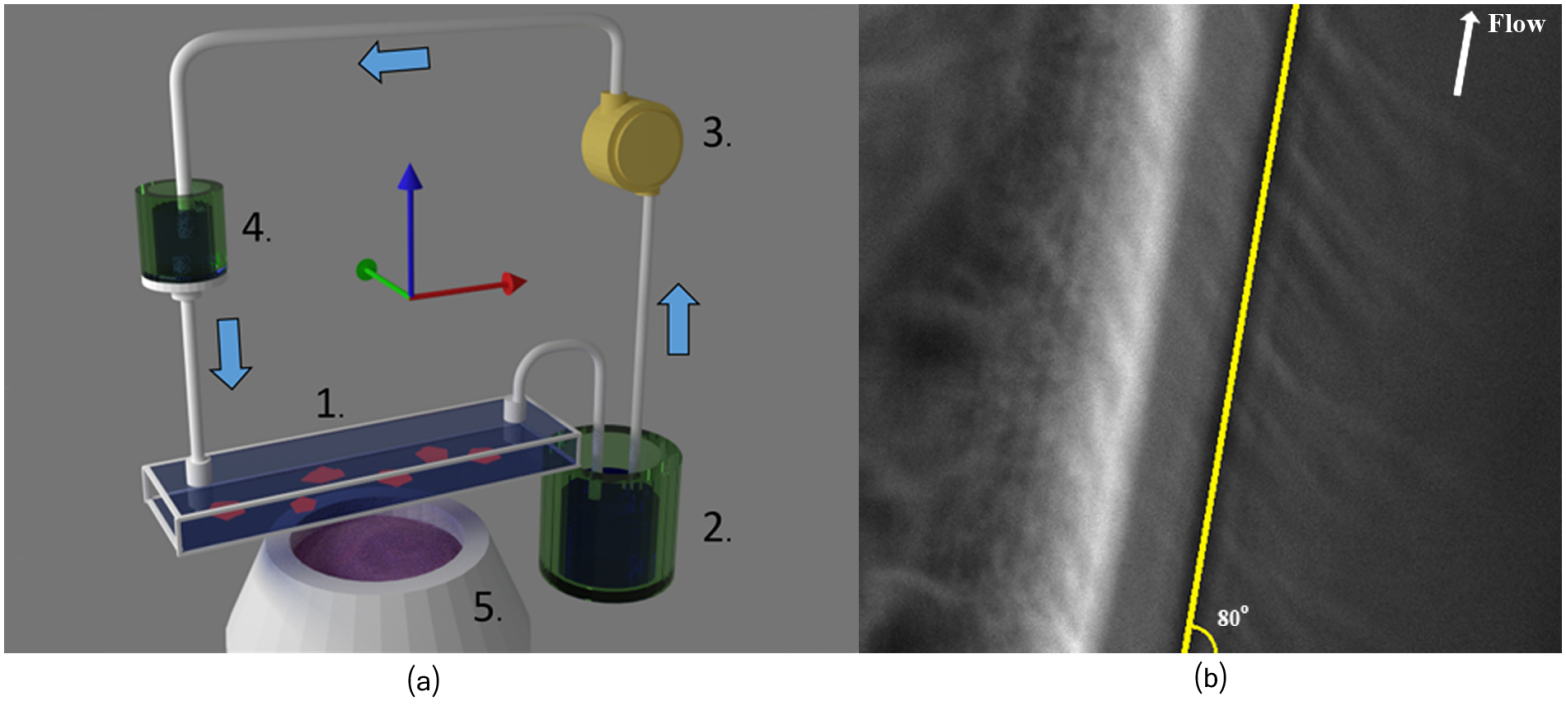
Fluid shear stress induction. (a) Schematics of the fluidic system we used to exert a constant shear stress on osteoblasts. 1: Ibidi culture chamber with osteoblasts attached to the lower plate (not depicted). 2: Container located under the culture chamber. 3: Peristaltic pump. 4: Container located above the culture chamber. Its position above the culture chamber permits to adjust the flow rate. 5: Microscope and camera (not shown). (b) Image of the culturing chamber showing the border of the micro-fluidic chip, and the direction of the shear stress flow at ≈ 80°.

The shear stress was induced by a fluidic system composed of two containers and a pump. This system, depicted in Fig 4a, permits a gravity driven constant flow of culture medium in the chamber containing the osteoblasts.

The flow rate was adjusted by setting the container (see Fig 4a nb 4) at the appropriate height above the osteoblasts. In our case, it provided a shear stress between 9 and 12 dyn/cm^2^. This value corresponds roughly to the one to which endothelial cells are exposed to in our arteries. The direction of the shear stress flow is estimated around ≈ 80° as illustrated in Fig 4b.

### Actin Staining of Osteoblasts

After stress exposure for 4 hours, the cells were rinsed in phosphate saline buffer (PBS) and chemically fixed with 4% paraformaldehyde (PFA) for 15 minutes. Before immunostaining the cells were rinsed twice in PBS. Eventually, the fixed cells were incubated with PBS 0.2% Triton X100 for 20 minutes, and exposed to Alexa Fluor 568 phalloidin (Molecular Probes) for 1 hour at room temperature. Finally the cells were rinsed again with PBS before a quick water rinse and coverage with cover glass (by using polyvinylalcohol (Sigma)). A static osteoblast culture, in the same ibidi device, was performed and actin stained as control.

### Osteoblasts Imaging

Imaging was performed with an inverted Axiovert 200M system with a 40x Plan-Neofluor (Carl Zeiss, Oberkochen, Germany). We used a motorized platform (MS-2000, Applied Scientific Instrumentation, with NanoDrive controller; Mad City Labs, Madison, WI) to scan the sample. Acquisition was performed using a CoolSnap HQ2 camera (PhotoMetrics, Tucson, AZ) and the Multi-Dimensional Acquisition module of the software Metamorph (Molecular Devices, Sunnyvale, CA).

### Image Decomposition

The problem of separating an image into different semantic constituent parts, ‘textures’ and ‘cartoon’, can be addressed by several approaches. Variational calculus [24, 25], and sparse multi-source separation [23] are among the most popular methods and their success varies depending on the image nature. In this work, we follow the Starck’s [23] image decomposition which is based on the Basis-Pursuit denoising (BPDN) algorithm. The basic idea behind this algorithm is to choose two appropriate dictionaries, one for the representation of ‘textures’, and the other for the ‘cartoon’ parts. Both dictionaries are to be designed such that each leads to sparse representations over the images it is serving, while yielding non-sparse representations on the other content type.

In our problem, we model an imaged (actin) cytoskeleton *f* as a combination of two sources plus some additive noise:

$$\begin{array}{c} f=u_{f}+v_{a}+\eta \end{array}$$ (1)

being *v*_*a*_ the background artifacts related content, and *u*_*f*_ the filamentous elements; the noise is represented by *η*. In [23] the sparse source separation problem, for the image *f*, has been defined as:

$$\begin{array}{ccc} \min_{\lambda_{a},\lambda_{f}} & & \|f-D_{a}\lambda_{a}-D_{f}\lambda_{f}{\|}_{2}^{2}+\gamma \|\lambda_{a}{\|}_{p}+\gamma \|\lambda_{f}{\|}_{p}+\delta (\nabla \lambda_{a}+\|\lambda_{a}{\|}_{1}) \end{array}$$ (2)

with *γ* > 0, *δ* ≥ 0 and *p* ∈ {0, 1}. The obtained sparse coefficients λ_*f*_ provides the fibers-related content *u*_*f*_ = *D*_*f*_ λ_*f*_, referred to as fibers image in the rest of the paper, and λ_*a*_ provides the artifacts related content *u*_*a*_ = *D*_*a*_ λ_*a*_, referred to as artifacts image; the norm *p* ∈ {0, 1} determines the reguralizer type and the parameter *γ* regulates the solution coefficients. The reconstructed image $\hat{f}=u_{f}+v_{a}$ is an approximation of *f* involving artifacts-related and fiber-related dictionaries, *D*_*a*_ and *D*_*f*_ respectively; the reminder $f-\hat{f}$ is usually related to noise *η*.

The definition of the dictionaries *D*_*a*_ and *D*_*f*_ is very much related to the nature of the different contents present in the image, therefore we considered different dictionaries based on fast transforms. For the artifacts related content we used an undecimated wavelet transform (UDWT) for modeling the dictionary *D*_*a*_, whereas for modeling the fibers dictionary *D*_*f*_, we used the curvelets transform. The latter being, as discussed in the introduction section, a natural choice for modeling curvilinear structures, while the wavelets transform allows modeling artifacts present in the images. Note that, in [23] the image component has been modelled by ridgelets and the texture (artefact) component by a Discrete Cosine Transform (DCT). Ridgelets has been also used in [50] to represent global lines in images.

**Fig 5:**
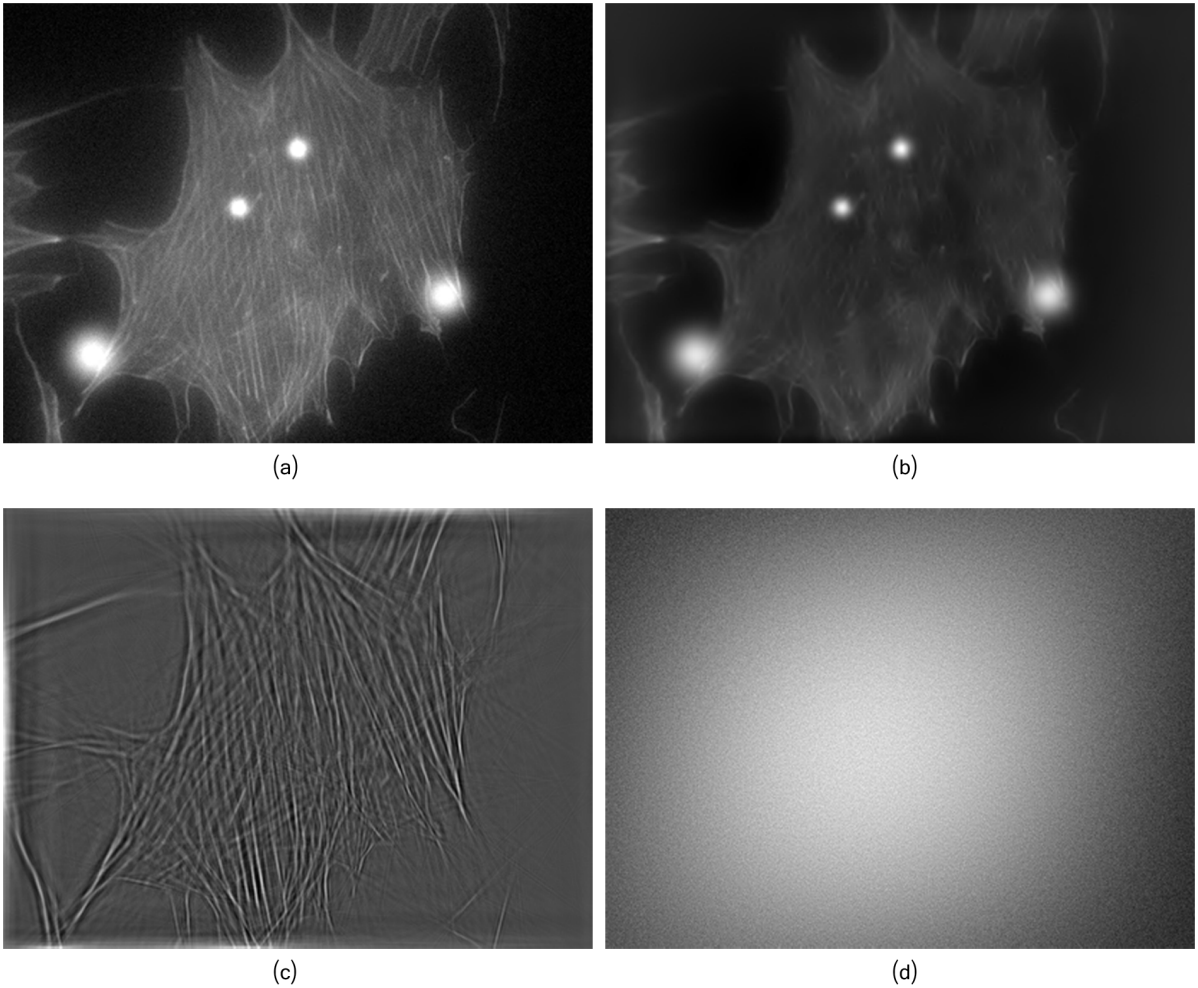
The decomposition process of the original image *f* into the artifacts image *v*_*a*_, the fibers image *u*_*f*_ and noise $f-\hat{f}$. (a) Image *f*. (b) Artifacts image *v*_*a*_. (c) Fibers image *u*_*f*_. (d) Reminder noise $f-\hat{f}$. Solution obtained after 100 iterations with *δ* = 3.

Our implementation of the image decomposition is based on the MCALab library provided in [54], running a maximum of 100 iterations. The parameter *γ* (in Eq 2) was linearly decremented during the iterations and initialized accordingly. For all reported experiments, we set *p* = 0 (in Eq 2), namely a *ℓ*_0_-norm for the model definition, and *δ* = 3. Results of the the image decomposition are illustrated in Fig 5. We refer the reader to [50] for more details on the solution algorithm, including initialization and updates of the *γ* parameter (in Eq 2), and to the Supporting Information (SN. 1.1 in S1 Text) for a discussion on the impact of the different parameters values on the decomposition results.

#### Filaments enhancement

**Fig 6:**
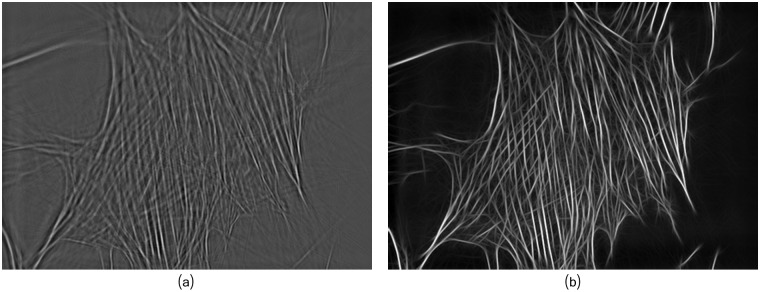
Fibers enhancement of the fibers image *u*_*f*_ provides sharpened filaments network. (a) Fibers image *u*_*f*_ after sparse multi-source separation. (b) Filaments enhanced image, *u*_*E*_, obtained with *σ* = 1.0, *β* = 10.0 and *σ*_*dg*_ = 10.0, for the Gaussian, Laplace and directional Gaussian, respectively.

Once the fibers related image (*u*_*f*_) has been properly separated from other artifacts, the filaments are enhanced in order to make the fibers more visible with respect to the background. Likewise in [21], we used a sequence of filters to enhance the filaments, consisting in: a Gaussian filter (a convolution operator that uses a kernel representing the shape of a Gaussian, parameterized by *σ* the standard deviation of the distribution); followed by a Laplace filter, with the following kernel:

$$\begin{array}{c} L=\beta \times \left[\begin{array}{ccc} 0 & -1 & 0\\ -1 & -4 & -1\\ 0 & -1 & 0 \end{array}\right] \end{array}$$ (3)

where β∈R is a parameter that we usually set as 0 < *β* ≤ 10.0. In most of our experiments we set *β* = 10. The result of such operator is a sharpened image highlighting the edges. For the third preprocessing step, we apply a directional Gaussian filter, with a filter size of *r* = 2⌈3*σ*_*dg*_⌉ (next larger integer) [21] with *σ*_*dg*_ = 10.0 in most of our experiments. Fig 6 illustrates the results of this processing chain, denoted as *u*_*E*_, applied on the fibers image *u*_*f*_.

### Multi-scale Line Segmentation

**Fig 7:**
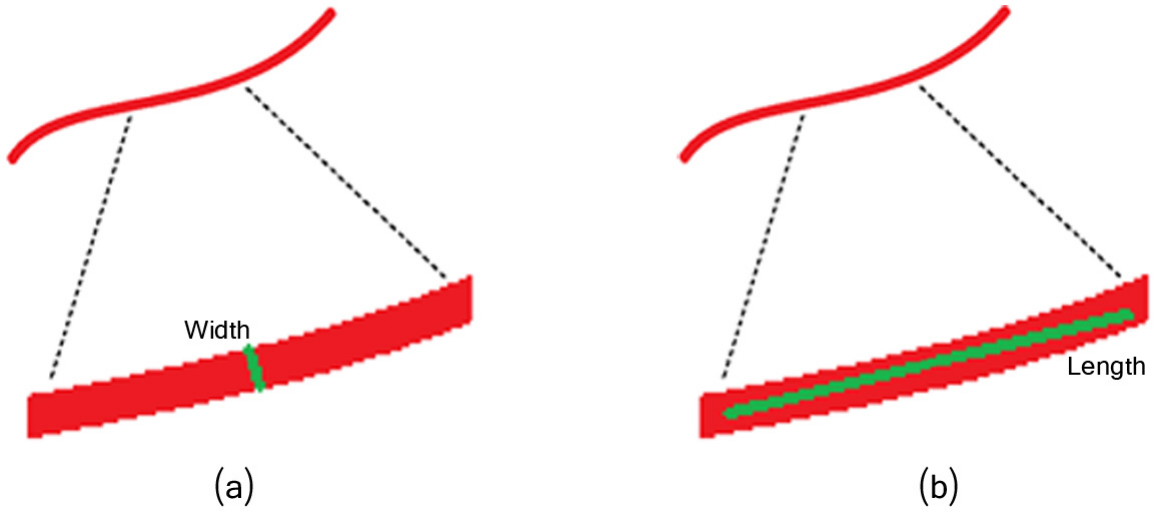
Linear response for width and length evaluation at certain orientation. (a) Width evaluation. (b) Length evaluation.

For the filaments network segmentation, we opted for a multi-scale line detector based on a structural element of different orientations and widths, representing the scales. The multi-scale line detector basically analyze each pixel’s neighborhood of *u*_*E*_ at different scales by evaluating (according to a score) if such pixel is part of a line of certain width. Such evaluation is performed by computing a ‘line response’ for the width evaluation, but also for a length evaluation that will be used in the next processing step for individual lines segments detection, as illustrated in Fig 7.

The line response takes place at each pixel in the image, in a (discrete) set of possible orientations between 0 and 180 degrees, considering linear elements of size *s* ∈ [1, *W*], with *W* the expected fibers width (this parameter is related to the magnification of the image). Then, the final multi-scale line response of each pixel (*x*, *y*) of the enhanced fibers image, *u*_*E*_, is provided as [34]:

$$\begin{array}{c} u_{G}\left(x,y\right)=\frac{1}{W+1}\sum_{s=1}^{W}\max_{\theta \in \{0,\cdots ,180\}}R\left(u_{E}\left(x,y\right);\theta ,s,W\right)\;\forall \left(x,y\right)\in \Omega \end{array}$$ (4)

where *R*(.), applied on the fibers enhanced image *u*_*E*_, provides a score indicating how likely is certain point to be the center of a line passing though it with a certain width and a certain direction, by analyzing the neighboring points. The reader is referred to [34] for the details on how *R*(.) is computed.

**Fig 8:**
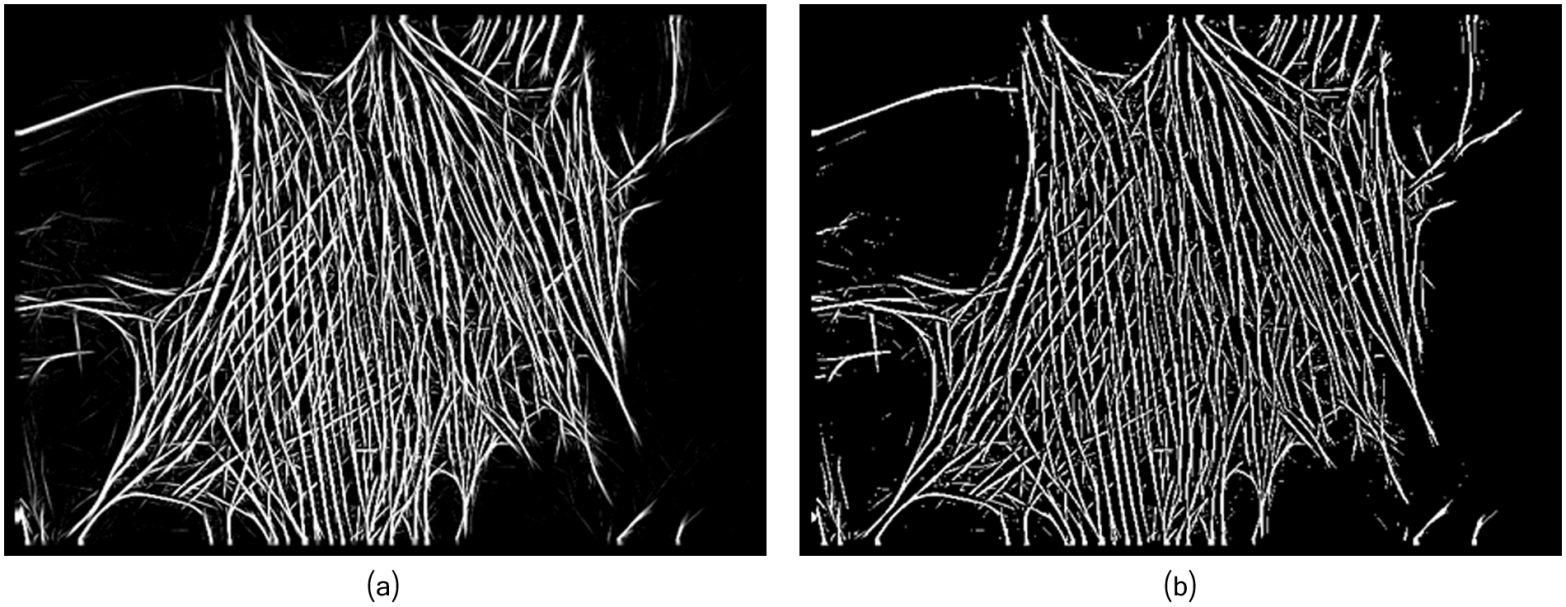
The binarization step provides the segmented filaments network. (a) Multi-scale linear response *u*_*G*_. (b) Binary image *u*_*B*_ obtained from *u*_*G*_. Width parameters set as *W* = 4.

In our implementation, based on the source code provided by [34], we retained the advantages of the multi-scale linear response image, *u*_*G*_, and at the same time introduced some modifications to obtain the final binary image, *u*_*B*_ of the fibers-related pixels. For the latter, we applied on the *u*_*G*_ gray-scale image a local thresholding algorithm to separate background and fibers pixels. For this purpose, we made use of the Wellner’s adaptive thresholding [55] were a median filtering provides an estimated local threshold. The final retained local threshold is a percentage *b* of the estimated threshold. The smaller *b* is the more line segments candidates are retained in the final binary image, representing a set of edge segments, which are clean, contiguous, i.e., connected, chains of edge pixels. Fig 8 illustrates the output of this step. Note that the obtained edges have a width larger than 1 pixel.

#### Line segmentation

From the obtained binary image we perform a line segmentation step that provides a list of straight line segments with fixed-length, denoted by (*F*, *θ*). Each line segment is represented by its parametric equation. The basic idea of the algorithm is to walk over the pixels in sequence, and fit lines to the pixels using the least squares line fitting method until the error exceeds a certain threshold, i.e. 1 pixel error. When the error exceeds this threshold, we generate a new line segment. The line segment is kept if its length is (at least) equal to *L*, a predefined parameter. In most of our experiments this parameter was set to *L* = 30.

### Filament Segment Merging—Fibers Extraction

**Fig 9:**
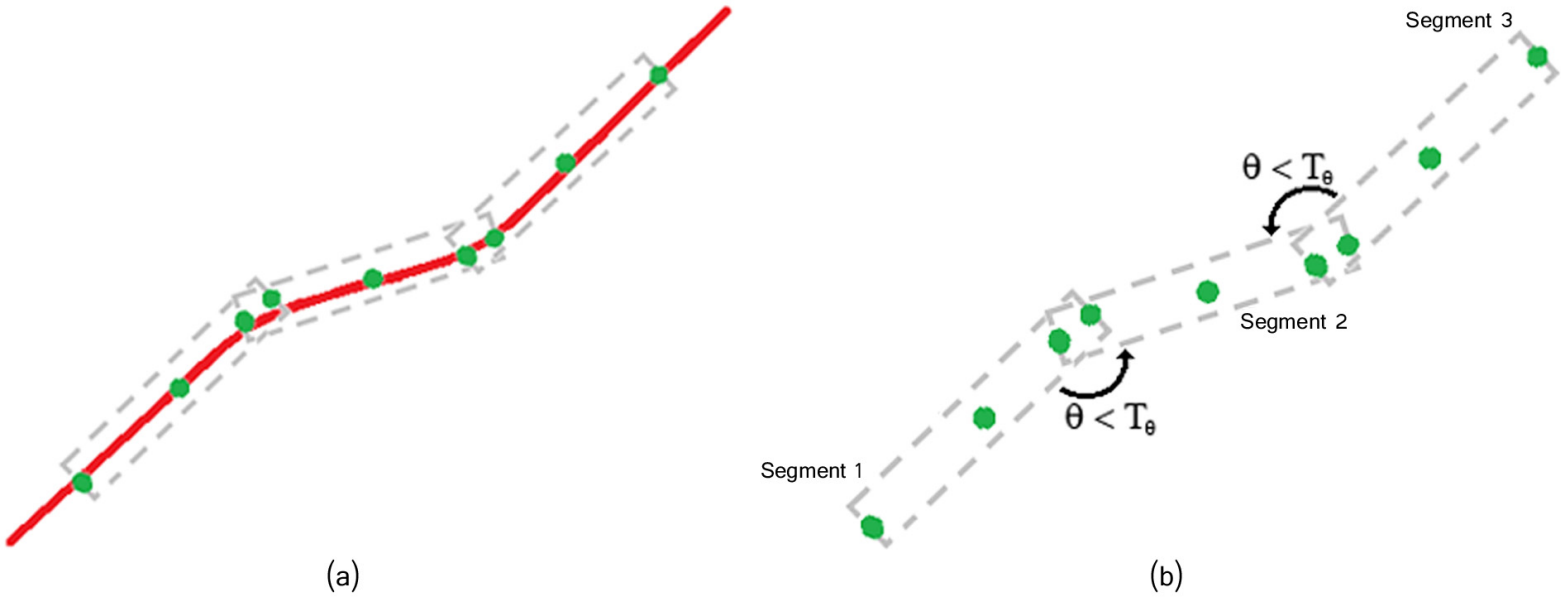
Fixed length segments are merged if they overlap and their angle difference is not too large *θ* < *T*_*θ*_. (a) Filament partitioned into fixed-length segments. (b) Overlapping and angle difference between segments.

Several complex approaches of line grouping, such as the on in [56], have been proposed within the computer vision community. However, for our purpose of fibers extraction, we developed a simple algorithm capable of iteratively extracting continuous linear segments (denoted as filament segments) by connecting the fixed-length segments extracted in the previous step. We first associate to a given filament segment all overlapping fixed-length segments oriented in the same direction (same *θ*). By repeating this process, all the overlapping segments with the same orientation will be combined into a longer straight-line segment. We then connect segments according to their orientation difference up to a ‘curvature’ threshold *θ* < *T*_*θ*_. In addition, when merging (i.e. connecting) the *k*-th and the *i*-th segments (*F*_*k*_⋃*F*_*i*_), we discard all the pixels that are beyond the connection (intersection) point. The above described procedure is detailed in Algorithm (1), and illustrated in Fig 9.

**Algorithm 1** Fibers extraction

 *θ*_*i*_: Indicates the orientation of extracted fiber *F*_*i*_

 *δ*(*F*_*i*_): Indicates the length of extracted fiber *F*_*i*_

 Input: *T*_*θ*_ as angle merging tolerance

 (*F*, *θ*)← List of fixed-length extracted segments tuples (*Line segmentation* section)

 **for** *ξ* = 0⋯*T*_*θ*_ **0**

  **for** *i* = 1⋯ **do**

$S\leftarrow \{k\in N^{+}\;|\;k>i,\;-\xi \leq \theta_{k}-\theta_{i}\leq \xi ,\;$ OverlapEndPoints(*F*_*k*_, *F*_*i*_)}

   **if** *S* ≠ ∅ **then**

    *k* ← *S*(1)

$\left(F_{k},\theta_{k}\right)\leftarrow \left(F_{k}⋃F_{i},\frac{\delta \left(F_{k}\right)\theta_{k}+\delta \left(F_{i}\right)\theta_{i}}{\delta \left(F_{k}\right)+\delta \left(F_{i}\right)}\right)$

   **end if**

  **end for**

 **end for**

 Output: list of individual fibers (*F*, *θ*)

 Note: *F*_*k*_⋃*F*_*i*_ combines the segments *F*_*k*_ and *F*_*i*_ discarding all the pixels located beyond the connection (intersection) point.

## Results and Discussion

**Fig 10:**
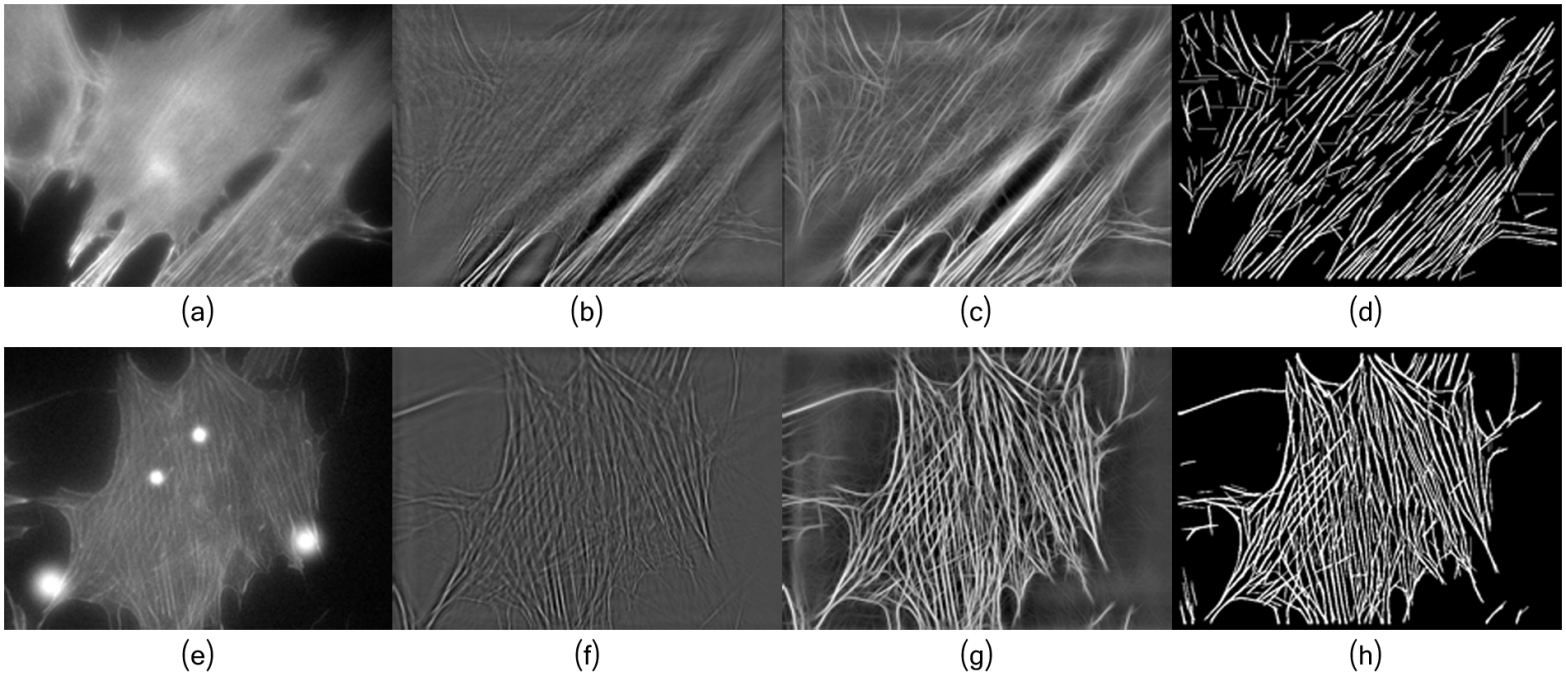
Individual filaments are extracted from the binarized image. (a) Input defocused image *f*. (b) Fibers image *u*_*f*_. (c) Enhanced fibers image *u*_*E*_. (d) Fibers from *u*_*B*_. (e) Input well focused image *x*. (f) Fiber image *u*_*f*_. (g) Enhanced fibers image *u*_*E*_. (h) Fibers from *u*_*B*_. For each processing stage, the parameters were set as in Figs 5, 6 and 8, respectively.

Fig 10 illustrates the results obtained at each step of the proposed framework (Fig 3). The robustness of the framework is qualitatively illustrated using a defocused image in Fig 10a and properly focused image in Fig 10e. In the defocused image of Fig 10a, only the lower part of the images exhibit clear (visually) filaments, and in other parts of the image the filaments are barely visible. As result of the image decomposition and multi-scale line segmentation, we obtain most of the filaments (Fig 10d). More details on the different steps and the impact of their parameters stetting are given in S1 Text.

In order to qualitatively and quantitatively assess the performances of the proposed framework, we considered two datasets. The first dataset, from [21], consist in a benchmark of 10 simulated images (referred to as *S*_1_, …, *S*_10_) and one real specimen cytoskeleton image (referred to as B2). The second dataset consist in 68 images of osteoblast cells (MC3T3-E1 cell line from mouse) grown in the two different conditions, static (control) and fluid shear stress, as described in section *Materials and Methods*.

### Qualitative Validation

#### Cytoskeleton image B2

**Fig 11:**
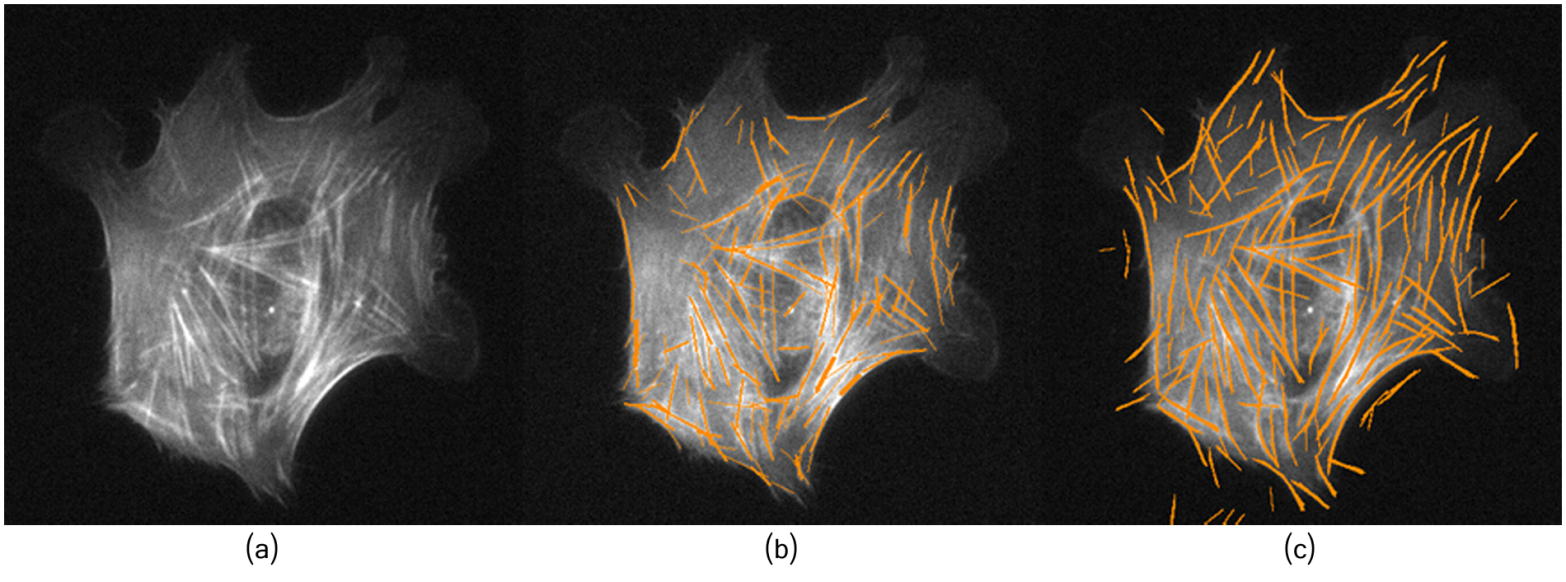
The proposed framework extract a higher number of filaments. (a) Original image B2. (b) Method of [21]. (c) Proposed framework with *W* = 4 and *L* = 30.

**Fig 12:**
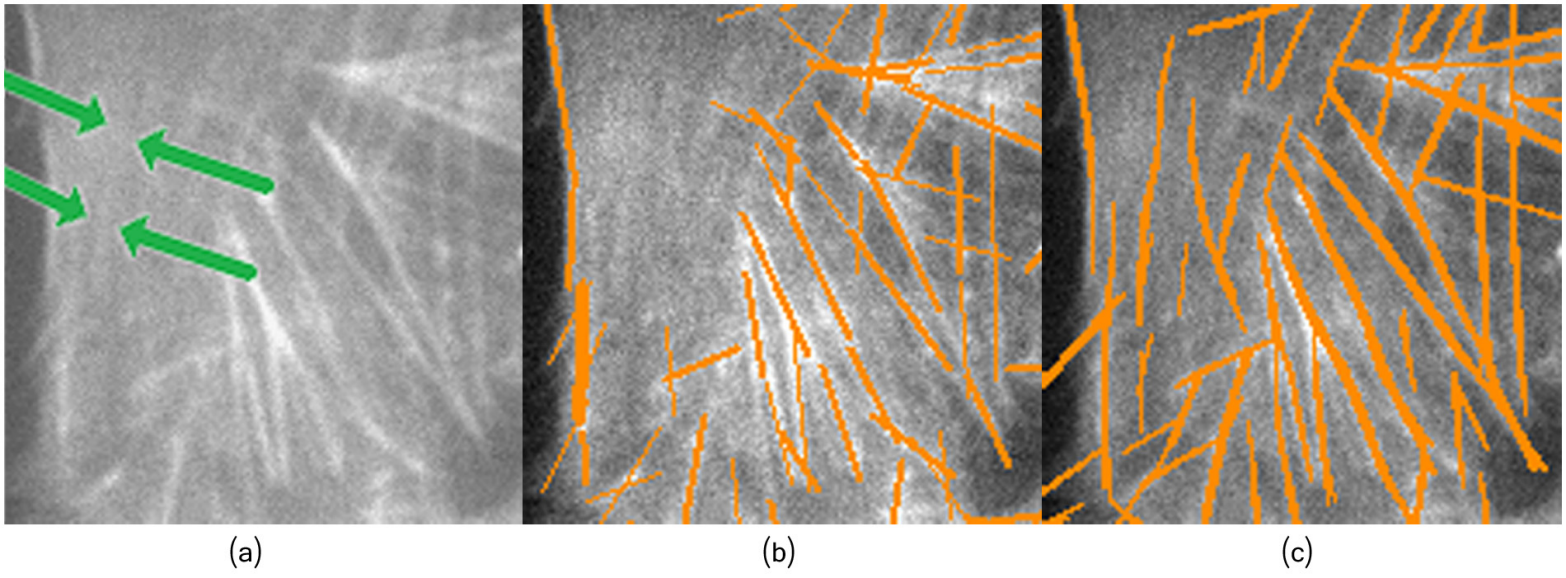
Zoomed area highlighting blurred filaments detected by the proposed framework. (a) Zoomed image B2. (b) Method of [21]. (c) Proposed framework.

From the data provided by [21], we selected the image of Fig 11a (referred to as B2). This challenging image has been selected as it exhibits highly blurred regions, with curvilinear filaments in addition to quasi-straight ones. Fig 11 illustrate the extracted filaments obtained using the proposed framework, compared to the method of [21]. A zoom-in of a very blurred image area is also given in Fig 12, where filaments indicated by the green arrows were missed by the method of [21], whereas the proposed framework succeeded to detect them.

**Fig 13:**
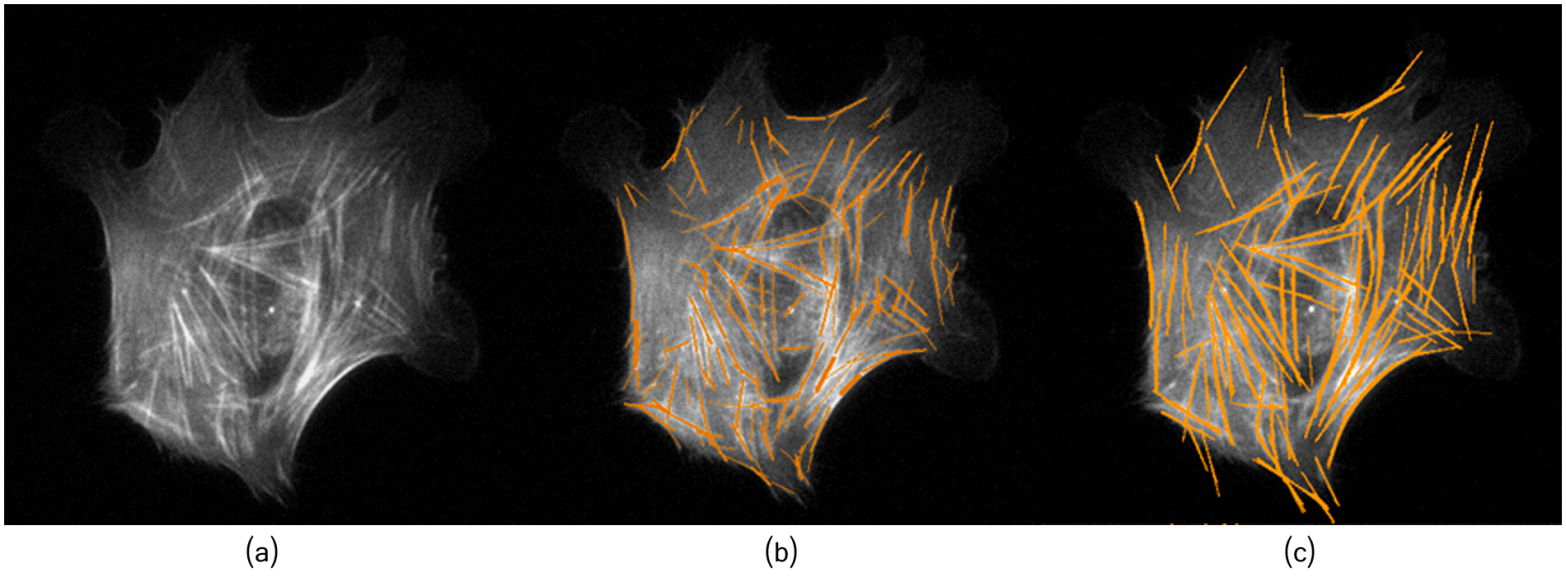
Filaments tracing. (a) Original image B2. (b) Method of [21]. (c) Proposed framework with *W* = 8 and *L* = 60.

The parameters *W* and *L* play an important role in reducing false positives. Note that results in Fig 11c exhibit some false positives, while in Fig 13c most of the filaments have been extracted with less number of false positives.

#### Peri-nuclear zone

**Fig 14:**
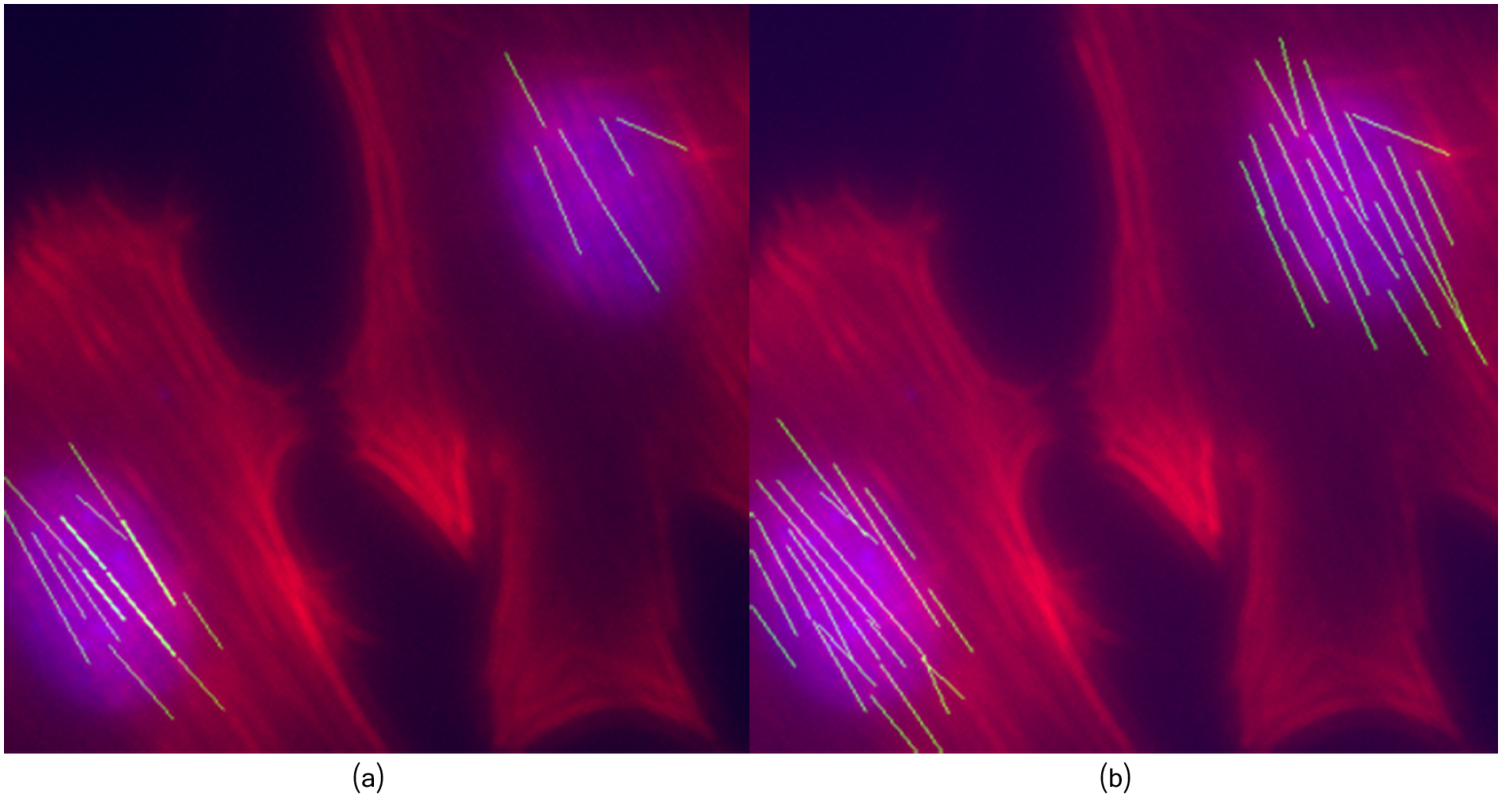
Extracted fibers in the peri-nuclear area. (a) Method of [21]. (b) Proposed framework.

The central role of actin cap in cells mechanosensing and mechanotransduction has been recently discovered [11, 12, 57, 58]. Thus, a proper filaments extraction on this area can be important to detect significant changes as result of mechanical stimulation. Images of actin filaments in the peri-nuclear zone are likely to exhibit some degree of blurring. Therefore, we assessed the framework performance in the peri-nuclear area of osteoblasts grown under fluid shear stress conditions. Fig 14 illustrates some of the extracted fibers using our method, compared to the results obtained using the method of [21].

As can be seen, compared to [21], the proposed framework is able to find most of the visible filaments. Although both methods detected several filaments in the bottom-left cell, only the proposed approach detected most of the filaments located in the peri-nuclear area of the top-right cell. Indeed, thanks to the image decomposition approach, several blurred quasi-straight clues were associated as part of filaments by the proposed framework.

### Quantitative Evaluation

#### Simulated filament networks

In order to quantitatively measure the model performance, we considered a set of 10 simulated images available in the benchmark database of [21], which consist in a set of filaments of different widths and lengths, corrupted with noise.

From the experimental results we computed the model accuracy (Acc), sensitivity (Sn) and specificity (Sp), defined as:

$$\text{Acc}=\frac{TP+TN}{TP+TN+FP+FN}$$ (5)

$$\text{Sn}=\frac{TP}{TP+FN}$$ (6)

$$\text{Sp}=\frac{TN}{TN+FP}$$ (7)

where *TP, TN, FP, FN* stands for:

- *TP*: pixels correctly identified as part of the filaments network
- *FP*: pixels wrongly identified as part of the filaments network
- *TN*: pixels correctly identified as not being part of the filaments network
- *FN*: pixels wrongly identified as not being part of the filaments network

**Fig 15:**
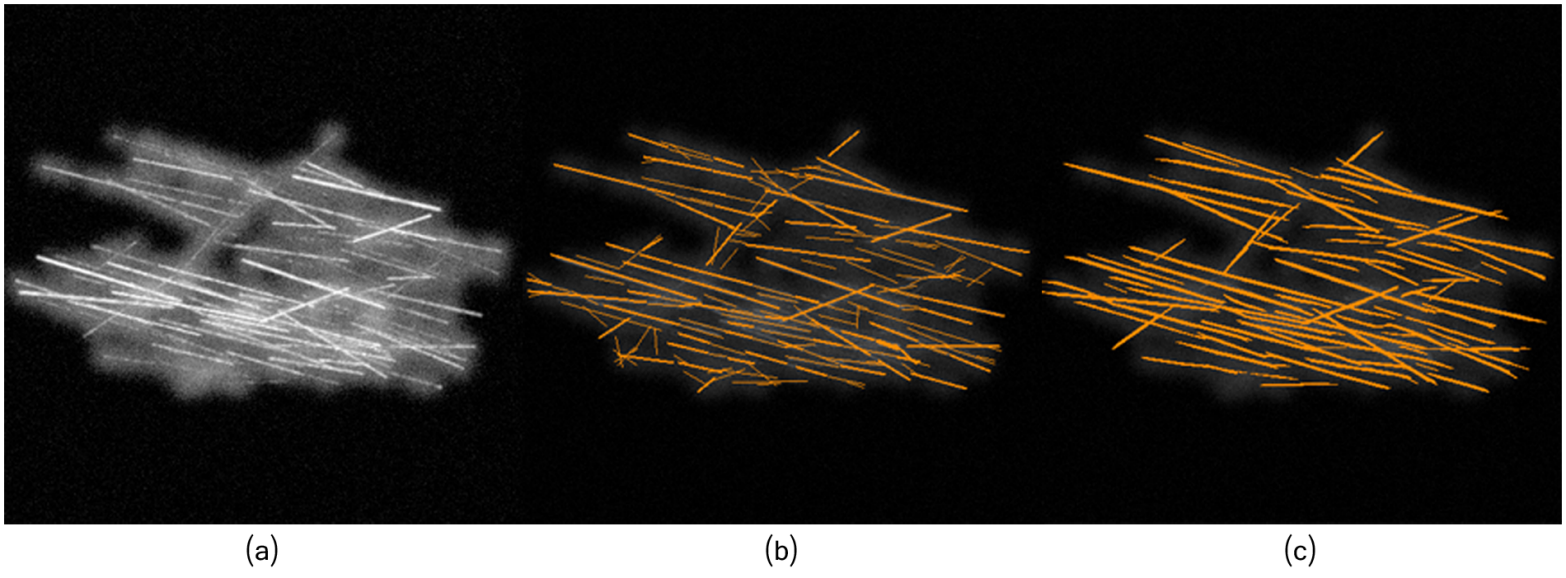
Most of errors accounted in the proposed framework consist of fibers longer than they should. (a) Simulated image (*S*_1_). (b) Method of [21]. (c) Proposed framework.

Results of the method in [21] were publicly available for the benchmark database, thus we only had to run experiments for the proposed method; Fig 15 illustrates both results in one of the simulated images of [21].

**Fig 16:**
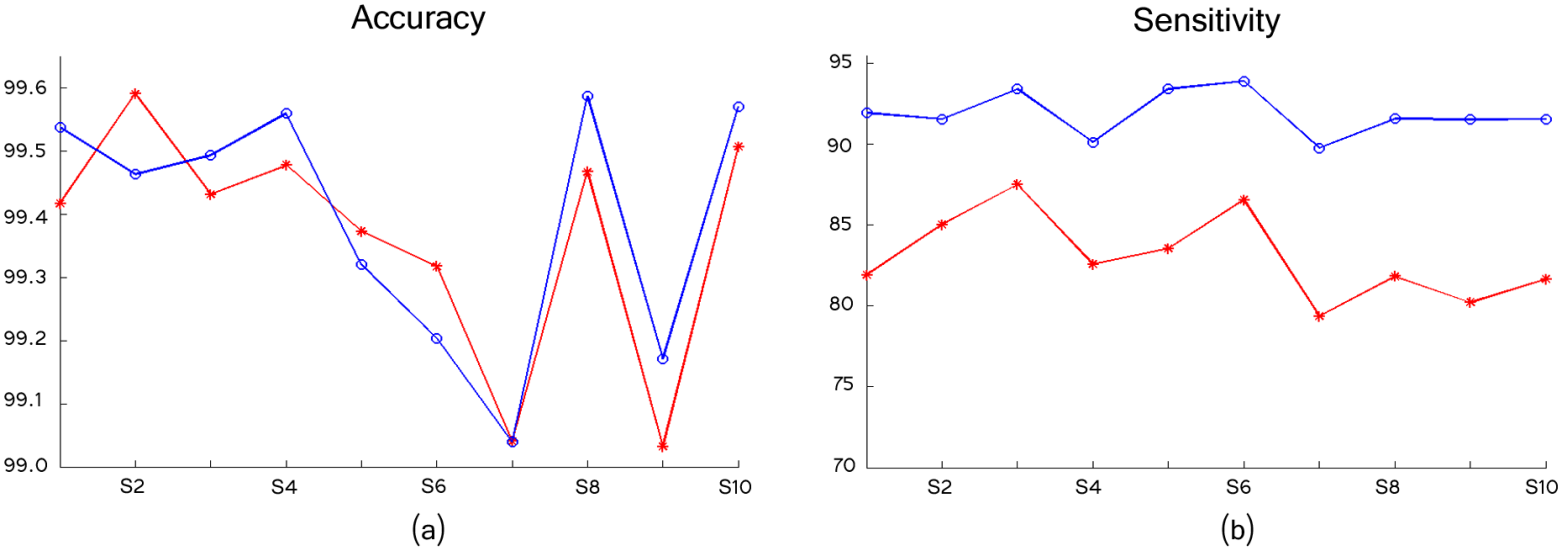
The models exhibit similar accuracy but a higher sensitivity, in 10 simulated images; *Stars* corresponds to method in [21] and *Circles* to the proposed framework results. (a) Accuracy. (b) Sensitivity.

The models accuracy and sensitivity are shown in Fig 16 for the 10 simulated images. As can be seen, both models perform similarly in terms of accuracy, although the proposed framework exhibited a little bit higher accuracy in 6 of the 10 images. In the proposed method, most of the incurred errors consist in false positive detections related to elongated fibers, and to a lesser extent some undetected thin filaments (false negatives).

However, the proposed model has a considerably higher sensitivity and a relatively similar (though lower) specificity, as detailed in Tables 1 and 2. This means that our model detected a higher number of filament pixels incurring in some false positive detections (mostly elongated fibers), while the model in [21] incurred in the same number of errors by failing to detect filaments.

**Table 1:** Sensitivity (Sn) comparison. Top-row: the method of [21]; Bottom-row: the proposed framework (PF).

	S1	S2	S3	S4	S5	S6	S7	S8	S9	S10
[21]	81.90	85.03	87.51	82.58	83.55	86.56	79.36	81.83	80.22	81.67
PF	91.95	91.57	93.42	90.12	93.43	93.91	89.78	91.57	91.53	91.57

**Table 2:** Specificity (Sp) comparison of method [21] in top-row and the proposed framework (PF) in bottom-row.

	S1	S2	S3	S4	S5	S6	S7	S8	S9	S10
[21]	99.88	99.92	99.82	99.91	99.90	99.85	99.92	99.92	99.90	99.93
PF	99.79	99.70	99.75	99.86	99.59	99.50	99.58	99.85	99.62	99.81

With respect to the specificity, there are no major differences, mostly due to the large number of background (negative) pixels present in the image; differences in sensitivity are significant.

#### Visually annotated osteoblasts images

**Fig 17:**
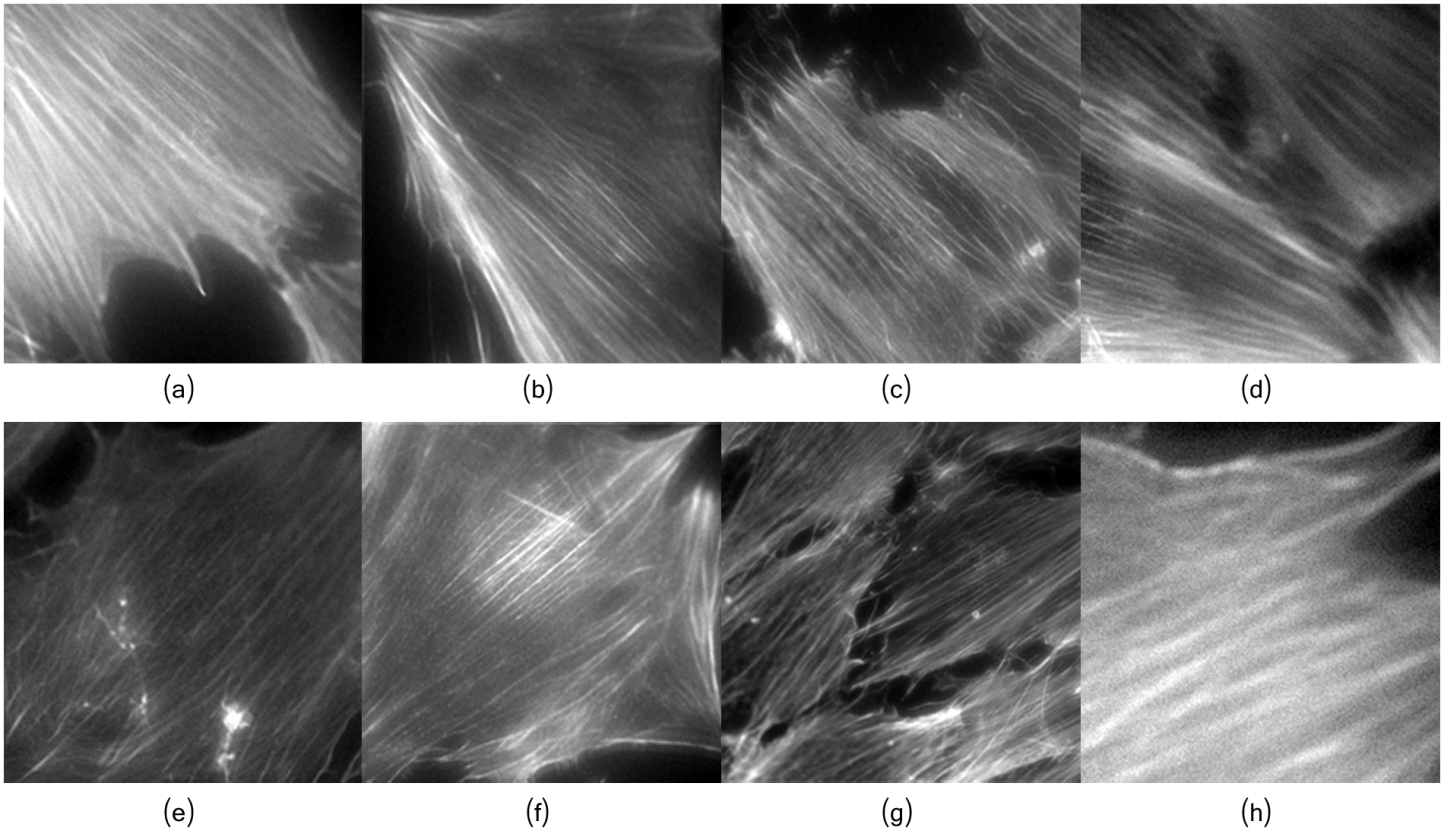
Visually annotated osteoblasts for orientation validation. (a-d) Images with fibers oriented to the left. (e-h) Images with fibers with fibers oriented to the right.

To asses the suitability of the proposed framework for filament’s orientation analysis, a set of osteoblats images/sub-images were visually annotated into upper-left oriented, upper-right oriented, or other orientation, according to the orientation of their fibers. From the annotated images, we randomly selected 18 images, 9 showing upper-left oriented fibers, denoted as Left-Set, and the other 9 showing upper-right fibers, denoted as Right-Set; Fig 17 depicts some images from each set. As it can be noticed, some of the selected images are heavily blurred, which can be handled by our framework (see Fig 10a–10d) thanks to the proposed image decomposition approach.

**Fig 18:**
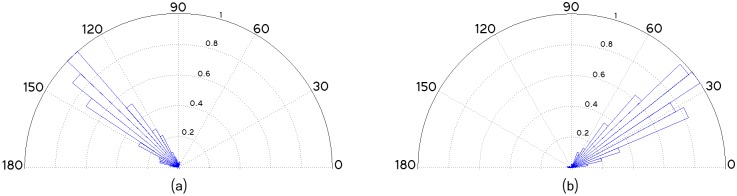
Normalized angular distribution of the Left-Set, and Right-Set, respectively, considering the horizontal axis of the image as reference. (a) Left-oriented fibers. (b) Right-oriented fibers.

The normalized angular distribution is depicted in Fig 18. It is computed by counting the number of detected individual fibers oriented toward each discrete direction, and dividing them by the maximum value. As it can be seen, the estimated fiber’s orientations correspond to the visually observed orientations. This confirms the suitability of the proposed framework for filament’s orientation analysis.

#### Effect of shear stress on actin filaments in osteoblasts

**Fig 19:**
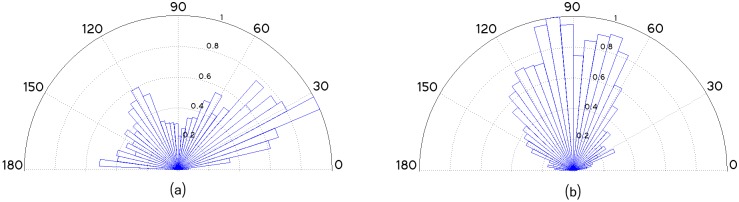
Normalized angular distribution of fibers grown in different stress conditions, taking as reference the horizontal axis of the image, and considering all images of each population. (a) Osteoblasts grown in static conditions. (b) Osteoblasts grown under fluid shear stress.

**Fig 20:**
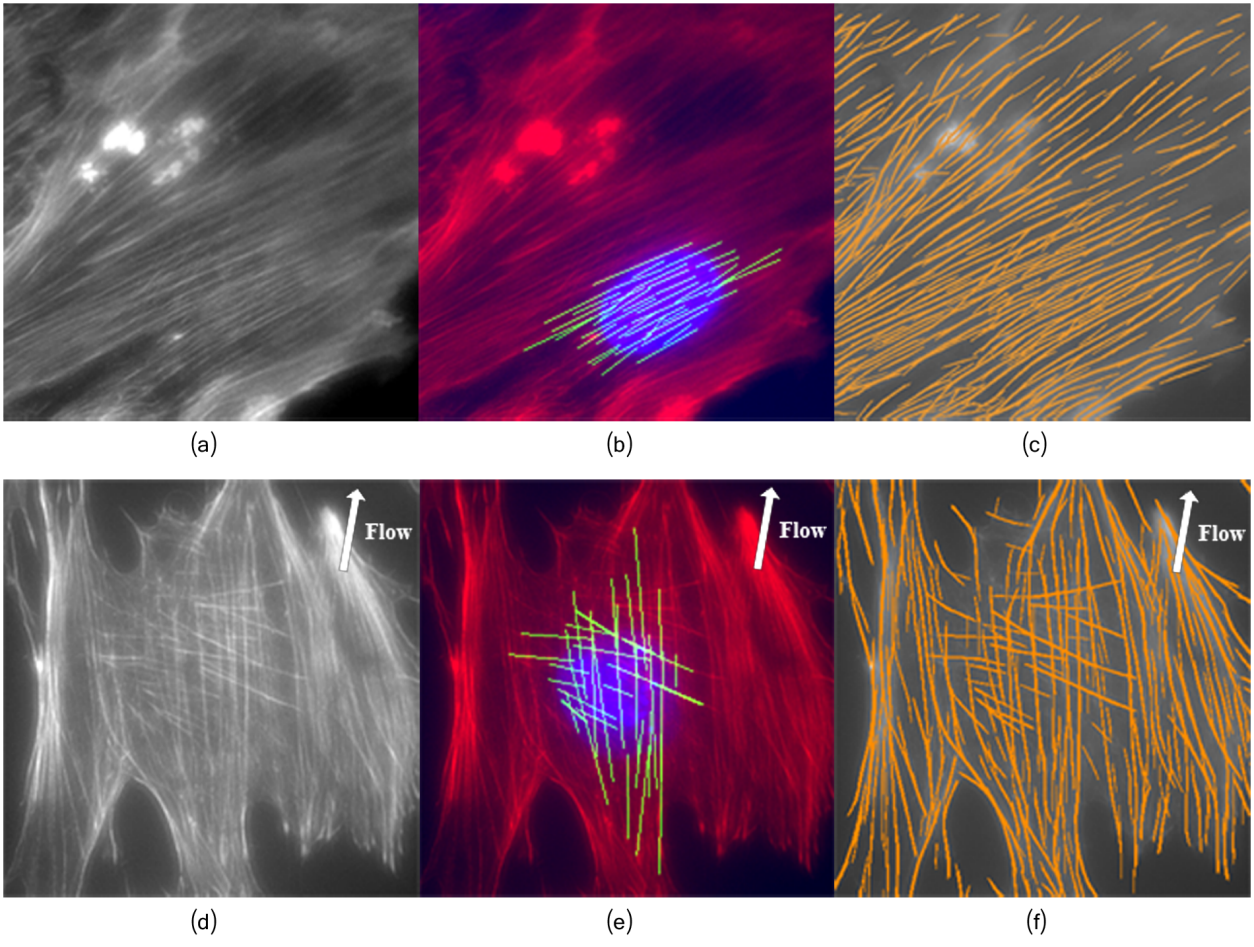
The proposed framework can properly extract highly overlapping fibers. (a) and (d) Osteoblast image. (b) and (e) Extracted fibers located near the nucleus. (c) and (f) Detected filaments network. Top row: Osteoblasts grown in normal conditions; Bottom row: Osteoblasts grown under fluid shear stress. The arrows depict the shear stress flow direction around ≈ 80° (Fig 4b).

In order to demonstrate the effectiveness of the proposed framework, the analysis of orientation, fibers quantification and length was performed in two population of osteoblasts grown in static conditions and under fluid shear stress (as described in Section Fluid shear stress induction). Application of shear stress to the cells is expected to cause reorientation of the actin stress fibers in a way that the filaments become nearly aligned with the shear stress flow direction [59, 60]. In our experiments, the osteoblast population grown under fluid shear stress contained a total of 32 images. The resultant filaments angular distribution over all the 32 images subjected to shear flow is illustrated in Fig 19. Fig 20 illustrates two osteoblast images and the detected fibers, in static and fluid- shear stress conditions.

To highlight the complexity of extracting the filaments in a cytoskeleton, we selected the image (Fig 20d) among the 32 images taken in the shear stress experiment. The results confirm that only a few filaments are oriented diagonally to the flow direction, and most of the stress fibers are aligned with the flow direction (see Fig 19e). The angular distribution of fibers orientation (with mean *μ* = 90.3 and variance *σ*^2^ = 1334.90) follows the almost vertical flow direction imposed to the shear stress cell culture (see Fig 4b), thus validating the accuracy of the method. The vast majority of the filaments located near the nucleus (Fig 20e) are well aligned with the flow. A smaller number of filaments, properly detected by our method, exhibited an orientation nearly diagonal with respect to the flow. In addition, most of these filaments are located in the peri-nuclear region thus likely related to the actin cap, suggesting their association to some strain avoidance response [11].

The filaments of the static (control) cells population (38 images) seems to cover the whole angular spectrum (with mean *μ* = 68.37 and variance *σ*^2^ = 3071.86), albeit a preference towards some specific angular orientations (Fig 19a) can be seen. This preference could reflect the orientation of the cells after hours of static growth in the channel.

The shear stress experiment exhibited a small variance value (*σ*^2^ = 1334.90) in the fibers orientation, which indicates that the vast majority of fibers are oriented towards the same direction. On the contrary, the variance of the static/control experiment (*σ*^2^ = 3071.86) is more than twice bigger compared to the shear stress experiment, evidencing that fibers are oriented towards several angular directions.

### Conclusions

The cytoskeleton plays an important role in numerous physiological and pathological processes, its morphological characteristics are therefore of prime importance to understand numerous basic cellular phenomena, such as cellular adaptation to physical or chemical stress. However, cytoskeleton quantification and analysis is far from being straightforward, and sophisticated algorithms are required to fulfill the task. In this work we present a processing framework that efficiently detects cytoskeletal fibers and quantifies its morphological characteristics such as the number of filaments it contains, their length and orientation. The proposed model was tested on images of osteoblasts cultivated in shear stress and static (control) conditions. The detection of highly oriented actin fibers in shear stress cultivated cells corroborates what one would expect in such a condition, i.e. an alignment of the fibers with the direction of the flow. In addition, our algorithm successfully detected the perinuclear actin cap, a structure difficult to detect by other state-of-the-art methods, and that seems to play an important role in mechano- transduction [11, 58]. It was also shown that the model separated filaments and imaging-related artifacts very efficiently, even in the presence of heavy blurring, a step that endowed the model with high sensitive detection capabilities. The proposed framework can be extended to extract 3D meshwork of actin filaments. Indeed, after the image decomposition and filament enhancements steps, a method such as the multiple Stretching Open Active Contours (SOACs) of [61] could be used for fibers tracing.

## Supporting Information

S1 Fig Effect of number of iteration on image decomposition results. (a) Fibers image *u*_*f*_ after 10 iterations. (b) Fibers image *u*_*f*_ after 100 iterations. (c) Fibers image *u*_*f*_ after 300 iterations. Those elements within the image that does not exhibit a filamentous geometry are progressively removed from the filaments component and put into the non-filament part.
(TIF)

S2 Fig Synthetic image. (a) Ground truth image *u*. (b) Generated Artifacts *v*. (c) Obtained synthetic image *f* = *u* + *v* + *η* with *η* a Gaussian noise *σ* = 0.04.
(TIF)

S3 FigImage decomposition of the synthetic image of S2c Fig, using 100 iterations and *γ* = 3.(a) Fibers image *u*_*f*_. (b) Artifacts image *v*_*a*_. (d) Reminder noise $f-\hat{f}$ with estimated noise level *σ* = 0.07.(TIF)

S4 Fig Different value of the filaments enhancement parameters (Gaussian filter, *σ*, Laplace filter, *β*, and linear Gaussian, *σ*_*dg*_) provides deferent filament saliencies. (a) *σ* = 0.5, *β* = 1.0 and *σ*_*dg*_ = 2.0. (b) *σ* = 1.0, *β* = 5.0 and *σ*_*dg*_ = 5.0. (c)*σ* = 1.0, *β* = 10.0 and *σ*_*dg*_ = 10.0. For visualization purposes, the brightness and contrast was regulated in exactly the same quantity on the three images.
(TIF)

S5 Fig Filament tracing. (a) Filaments enhancement of original image. (b) Filaments enhancement after image decomposition. (c) Multi-scale linear response (*W* = 2) followed by binarization step *b* = 10. (d) The line segmentation stage keeps only quasi-straight segments of a minimum length *L* = 30, discarding the others. (e) *W* = 2, *b* = 0.1 and *L* = 30. (f) *W* = 4, *b* = 0.1 and *L* = 30.
(TIF)

S6 Fig Straight line segments obtained with *T*_*θ*_ = 0. (a) Binary image. (b) Individual filaments. Only the 100 longest filaments are displayed. The different colors depict different orientations.
(TIF)

S7 Fig Extracted filaments with *T*_*θ*_ = 2. (a) Binary image. (b) Individual filaments. The different colors depict different orientations.
(TIF)

S1 Text Supplementary Notes. (PDF)
