# Supplementary figures and images for: A Robust Actin Filaments Image Analysis Framework
Source: PLoS Comput Biol. 2016 Aug 23;12(8):e1005063. doi: 10.1371/journal.pcbi.1005063 (PMC4995035; doi:10.1371/journal.pcbi.1005063)

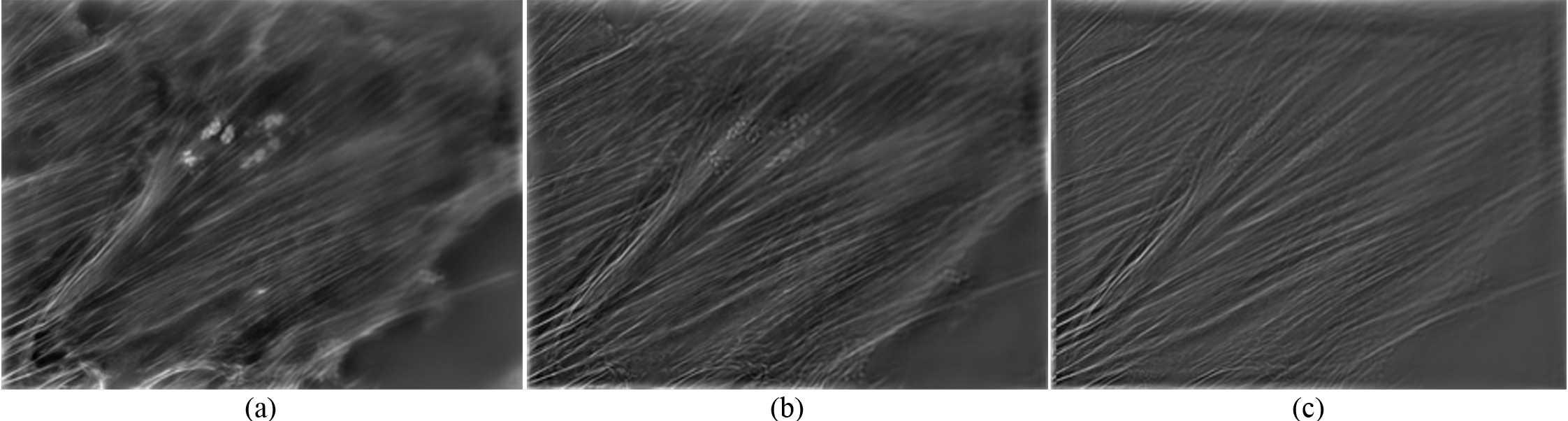

Supplement: S1 Fig — (a) Fibers image uf after 10 iterations. (b) Fibers image uf after 100 iterations. (c) Fibers image uf after 300 iterations. Those elements within the image that does not exhibit a filamentous geometry are progressively removed from the filaments component and put into the non-filament part. (TIF) [file pcbi.1005063.s001.tif]

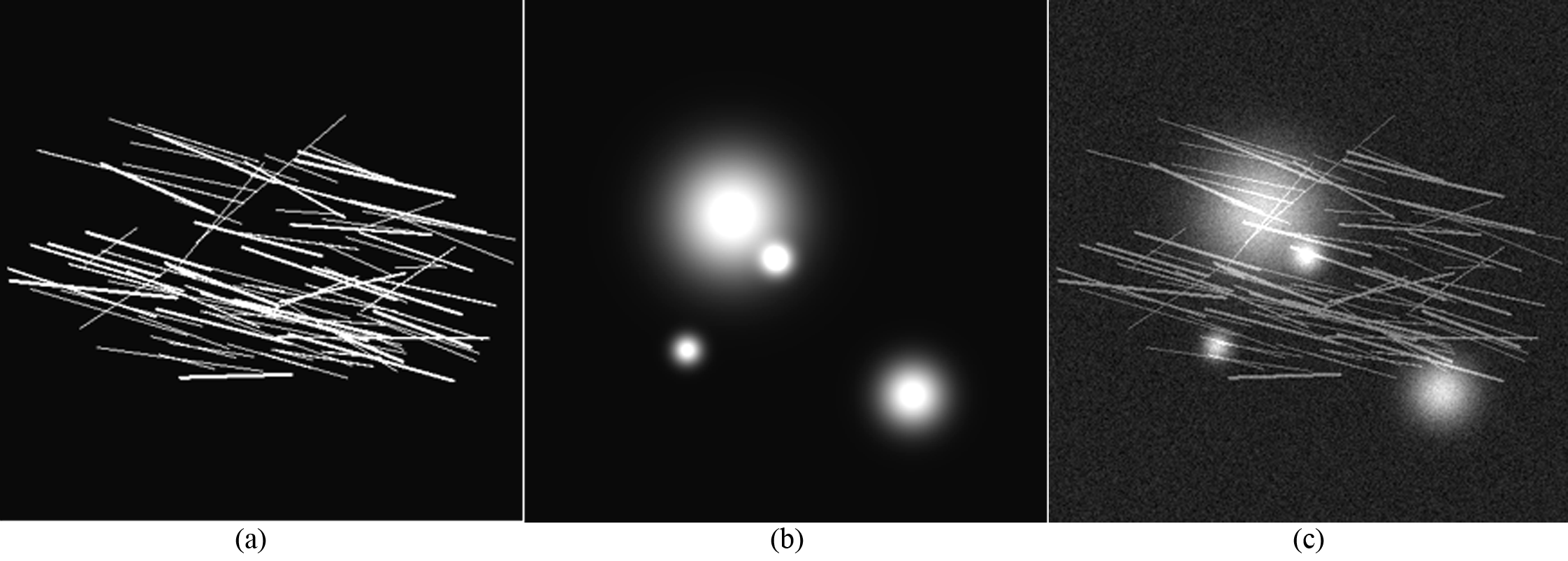

Supplement: S2 Fig — (a) Ground truth image u. (b) Generated Artifacts v. (c) Obtained synthetic image f = u + v + η with η a Gaussian noise σ = 0.04. (TIF) [file pcbi.1005063.s002.tif]

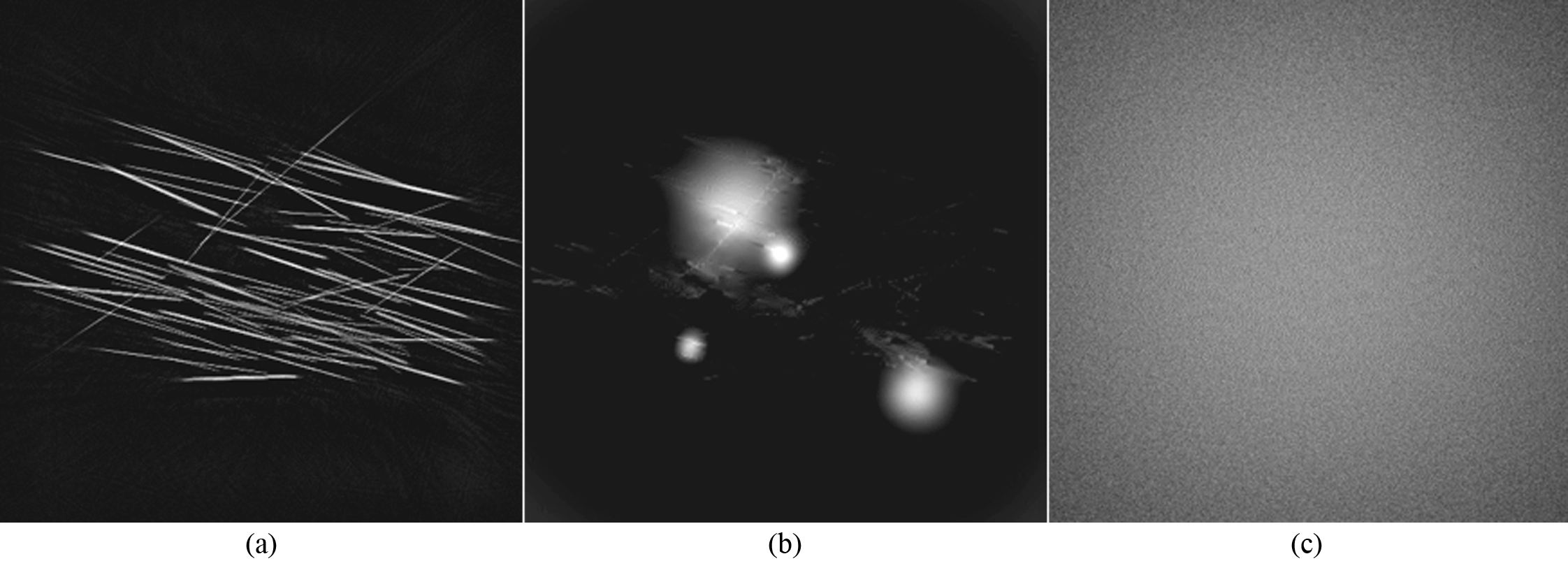

Supplement: S3 Fig — (a) Fibers image uf. (b) Artifacts image va. (d) Reminder noise f-f^ with estimated noise level σ = 0.07. (TIF) [file pcbi.1005063.s003.tif]

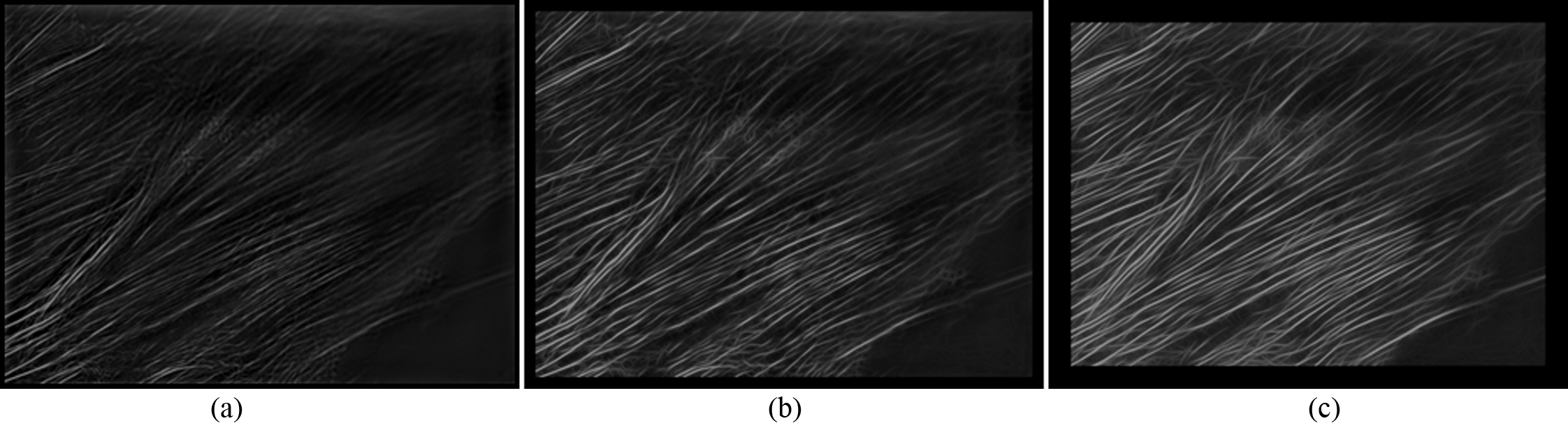

Supplement: S4 Fig — (a) σ = 0.5, β = 1.0 and σdg = 2.0. (b) σ = 1.0, β = 5.0 and σdg = 5.0. (c)σ = 1.0, β = 10.0 and σdg = 10.0. For visualization purposes, the brightness and contrast was regulated in exactly the same quantity on the three images. (TIF) [file pcbi.1005063.s004.tif]

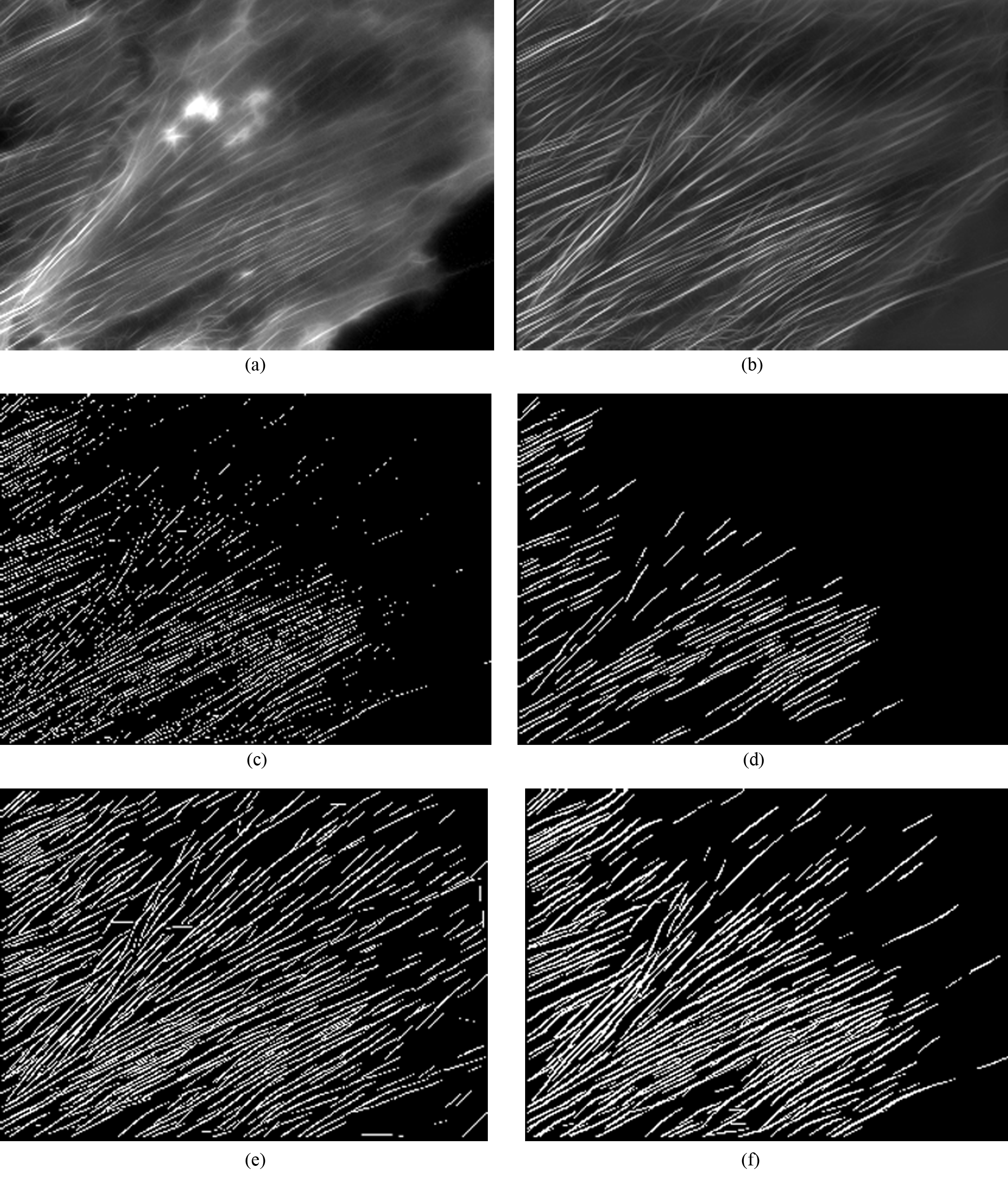

Supplement: S5 Fig — (a) Filaments enhancement of original image. (b) Filaments enhancement after image decomposition. (c) Multi-scale linear response (W = 2) followed by binarization step b = 10. (d) The line segmentation stage keeps only quasi-straight segments of a minimum length L = 30, discarding the others. (e) W = 2, b = 0.1 and L = 30. (f) W = 4, b = 0.1 and L = 30. (TIF) [file pcbi.1005063.s005.tif]

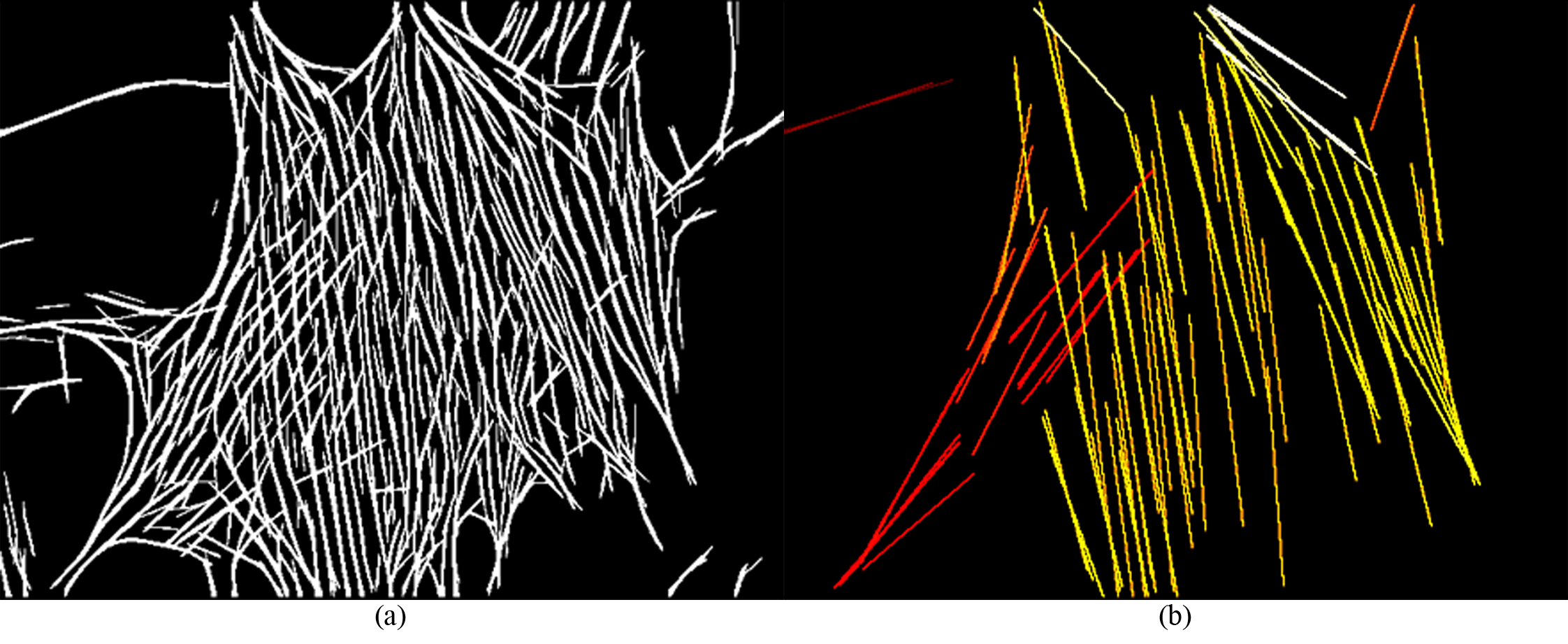

Supplement: S6 Fig — (a) Binary image. (b) Individual filaments. Only the 100 longest filaments are displayed. The different colors depict different orientations. (TIF) [file pcbi.1005063.s006.tif]

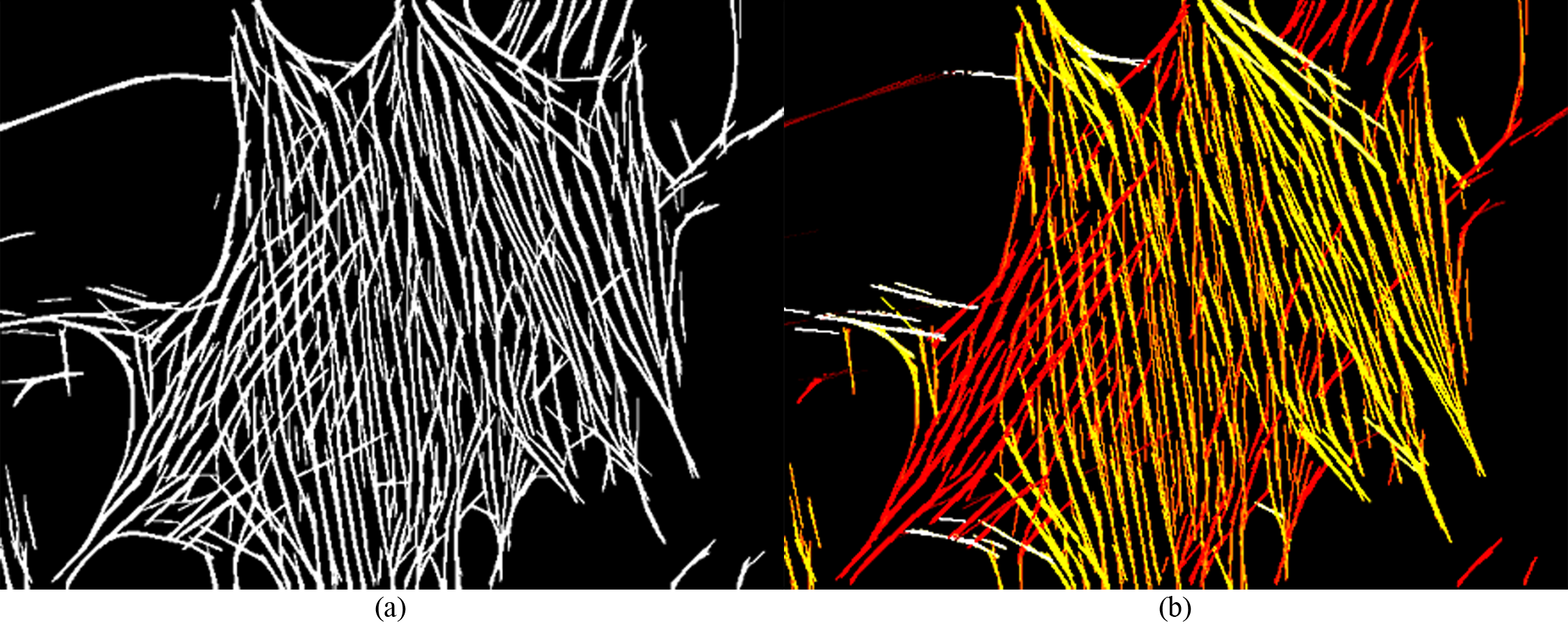

Supplement: S7 Fig — (a) Binary image. (b) Individual filaments. The different colors depict different orientations. (TIF) [file pcbi.1005063.s007.tif]
