# Supplementary material for: A Robust Actin Filaments Image Analysis Framework
Source: PLoS Comput Biol. 2016 Aug 23;12(8):e1005063. doi: 10.1371/journal.pcbi.1005063 (PMC4995035; doi:10.1371/journal.pcbi.1005063)
Supplement: S1 Text — (PDF) [file pcbi.1005063.s008.pdf]

Supplementary Material for the Article:
A robust actin filaments image analysis framework

Mitchel Alioscha-Perez, Carine Benadiba, Katty Goossens, Sandor Kasas, Giovanni Dietler, Ronnie Willaert, Hichem Sahli

1 Supplementary Note 1: Parameters settings of the processing steps

1.1 Image Decomposition.

Three parameters are involved in the image decomposition stage, namely: the norm $p \in \{0, 1\}$ which determines the regularizer type, the parameter γ which regulates the smoothness of solution coefficients, and δ which regulates the extra TV constraint on the artefact's component. The parameter γ is automatically estimated in the MCALab [1] implementation of the image decomposition algorithm of [2]. For all our experiments, the other parameters have been set as follows: the ℓ_0-norm for the model definition ($p = 0$), and the TV parameter δ has been empirically fixed to 3. Three additional parameters influence the output results: the level of the wavelet transform, the level of the curvelets transform, and the number of iteration of the optimization algorithm. For all our experiments we used 5 resolution levels for the wavelet and curvelets transforms.

With respect to the number of iterations, from Fig. S1 we can see that more iterations lead to better separation of the filamentous content from the artefact content, hence reproducing better the filamentous structures. However, at the cost of higher computational burden. Using the MCAlab [1] implementation, the total execution time for the decomposition of a 500×500 image, varies between 5 to 10 seconds per iteration; 100 iterations require approximately 8 minutes of execution time. For all our experiments the number of iterations was set to 100.

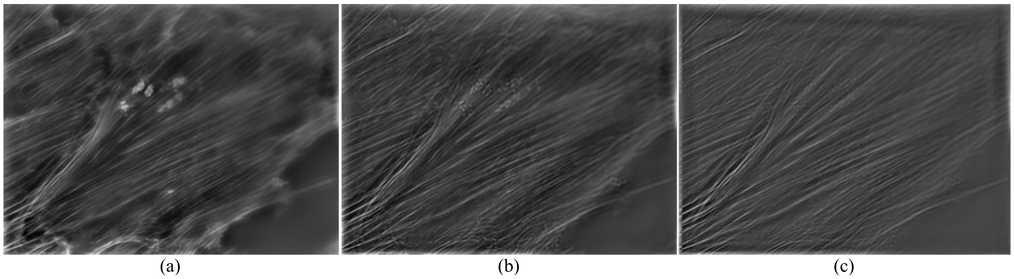

Figure S1. Effect of number of iteration on image decomposition results. (a) Fibers image u_f after 10 iterations. (b) Fibers image u_f after 100 iterations. (c) Fibers image u_f after 300 iterations. Those elements within the image that does not exhibit a filamentous geometry are progressively removed from the filaments component and put into the non-filament part.

To further illustrate the importance of the image decomposition in our proposed framework, we make use of a synthetically generated image composed of a ground truth image from [3] (see Fig. S2.a), to which we added the artifacts image of Fig. S2.b, and a Gaussian noise with $\sigma = 0.04$. The resulting synthetic image is given in Fig. S2.c.

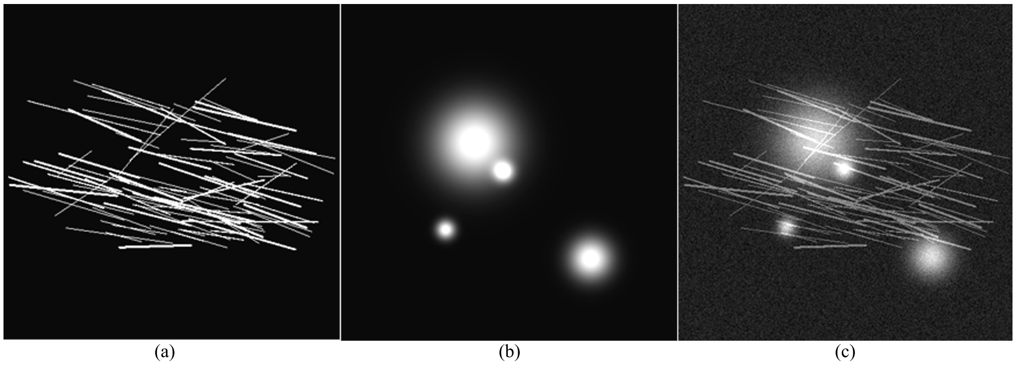

Figure S2. Synthetic image. (a) Ground truth image u. (b) Generated Artifacts v. (c) Obtained synthetic image $f = u + v + \eta$ with η a Gaussian noise $\sigma = 0.04$.

The reconstructed fibers component u_f, the artifacts part v_a, and the noise part are given in Fig. S3. As we can see, the separation is reproduced rather well.

1.2 Filaments enhancement.

For the filament enhancement we followed the approach in [3], though any other enhancement approach could be used in our framework. Indeed, the difference between the proposed framework and the one of [3] as well other approaches, is that we propose the image decomposition as a first stage to obtain a better fibers image, and the last stage of fibers tracing and merging (line segmentation). The filament enhancement

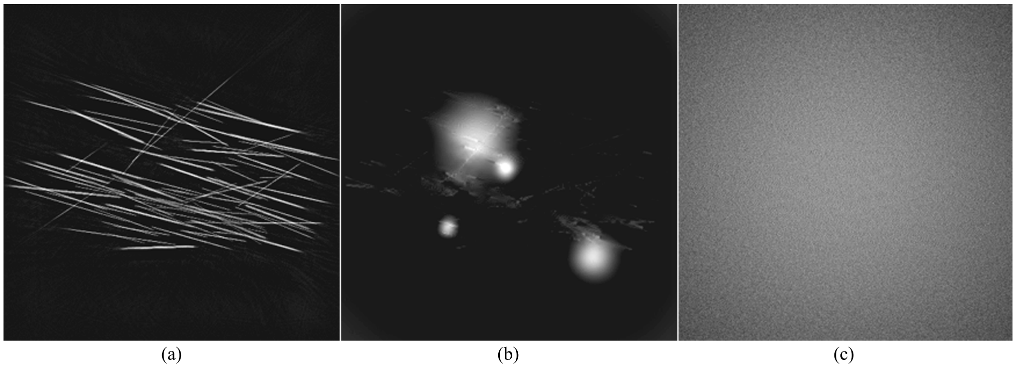

Figure S3. Image decomposition of the synthetic image of Fig. S2.c, using 100 iterations and $\gamma = 3$. (a) Fibers image u_f. (b) Artifacts image v_a. (d) Reminder noise $f - \hat{f}$ with estimated noise level $\sigma = 0.07$

stage provides a mean to connect non-continuous segments and enhance crossing-fibers without disturbing the saliency of any of them. The negative side of the used approach is that it elongates the segments making them to appear longer than they really are. The overall execution time of this stage (the three steps of Gaussian filtering, followed by a Laplace filtering and finally linear Gaussian filtering) is approximately 10s. The effect of the filtering parameters are illustrated in Fig. S4.

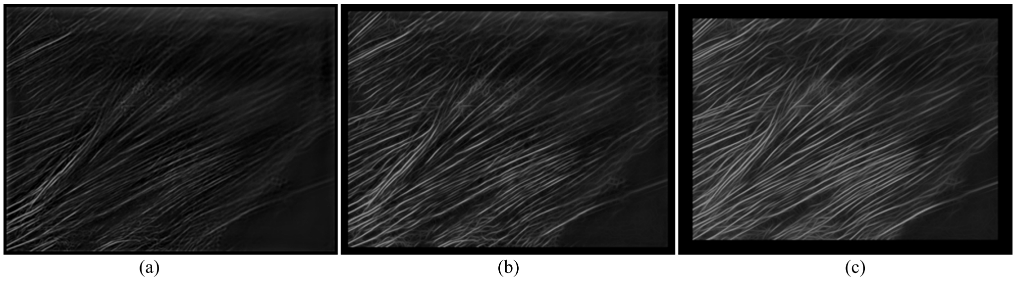

Figure S4. Different value of the filaments enhancement parameters (Gaussian filter, σ, Laplace filter, β, and linear Gaussian, σ_{dg}) provides deferent filament saliencies. (a) $\sigma = 0.5$, $\beta = 1.0$ and $\sigma_{dg} = 2.0$. (b) $\sigma = 1.0$, $\beta = 5.0$ and $\sigma_{dg} = 5.0$. (c) $\sigma = 1.0$, $\beta = 10.0$ and $\sigma_{dg} = 10.0$. For visualization purposes, the brightness and contrast was regulated in exactly the same quantity on the three images.

1.3 Mutli-scale line detector.

The multi-scale segmentation step requires prior information on the segments width W and length L, as well as the threshold b (percentage for the binarization). It usually takes less than a minute for an input image. Increasing the segment width will produce wider but also a less number of filaments. Setting the proper minimum length L is also very important in order to reduce/eliminate shorter non meaningful segments (see

Fig. S5).

1.4 Filament Segment Merging - Fibers extraction

The curvature threshold T_θ is defined by the user depending on the desired analysis. For extracting only straight lines T_θ is set to 0, while higher values such as $T_\theta \leq 2$, allows extracting filaments with some degree of curvature. Results obtained with $T_\theta = 0$ are illustrated in Fig. S6, where we displayed only the 100 longest extracted actin fibers to show that they are straight line segments. Fig. S7 illustrates all the detected filaments using as curvature threshold $T_\theta = 2$.

References

1. Signal and Image Decomposition and Inpainting v12.0. `https://fadili.users.greyc.fr/demos/WaveRestore/downloads/mcalab/Download.html`. (Accessed 30 September 2015).

2. Fadili, JM, Starck JL, Elad M, Donoho DL. MCALab: Reproducible research in signal and image decomposition and inpainting. Computing in Science and Engineering. 2010; 12(1): 44–63.

3. Eltzner B, Wollnik C, Gottschlich C, Huckemann S, Rehfeldt F. The Filament Sensor for Near Real-Time Detection of Cytoskeletal Fiber Structures. Plos One. 2015;10(5).

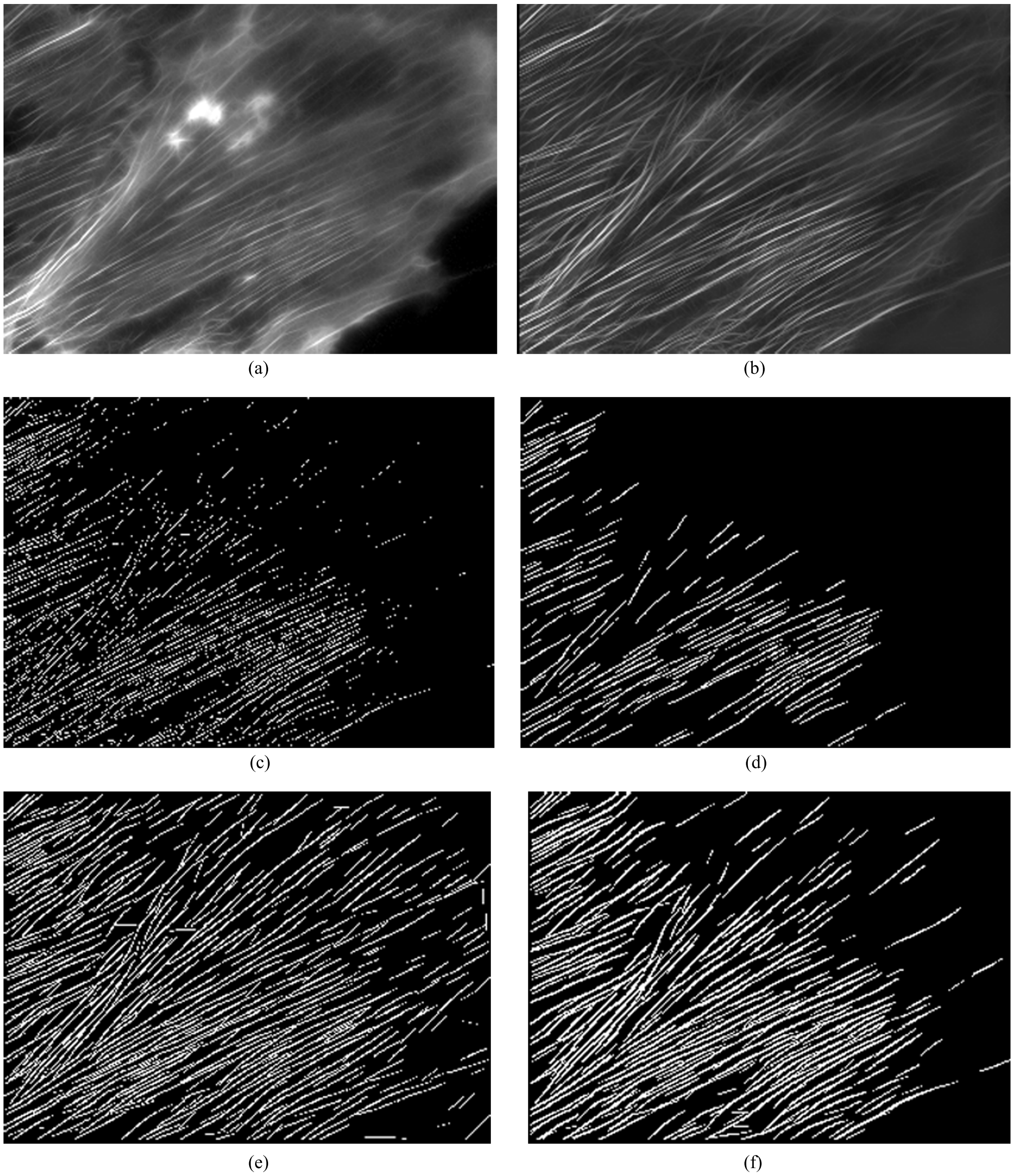

Figure S5. Filament tracing. (a) Filaments enhancement of original image. (b) Filaments enhancement after image decomposition. (c) Multi-scale linear response ($W = 2$) followed by binarization step $b = 10$. (d) The line segmentation stage keeps only quasi-straight segments of a minimum length $L = 30$, discarding the others. (e) $W = 2$, $b = 0.1$ and $L = 30$. (f) $W = 4$, $b = 0.1$ and $L = 30$.

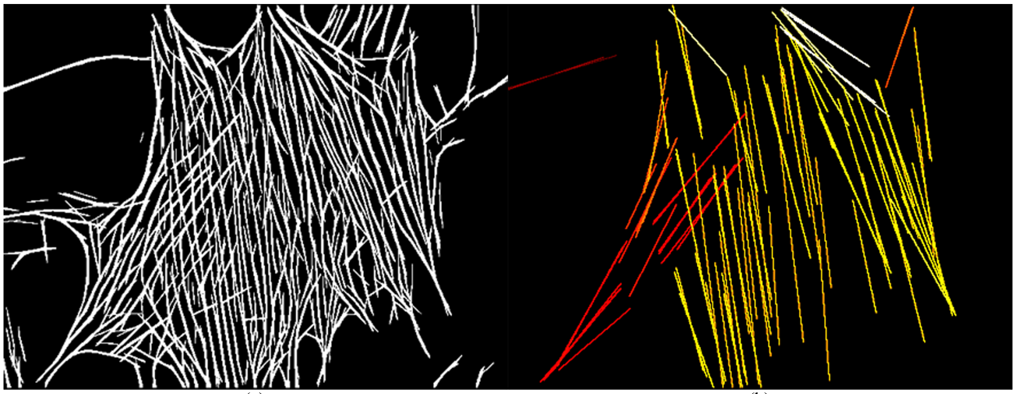

(a) (b)

Figure S6. Straight line segments obtained with $T_\theta = 0$. (a) Binary image. (b) Individual filaments. Only the 100 longest filaments are displayed. The different colors depict different orientations.

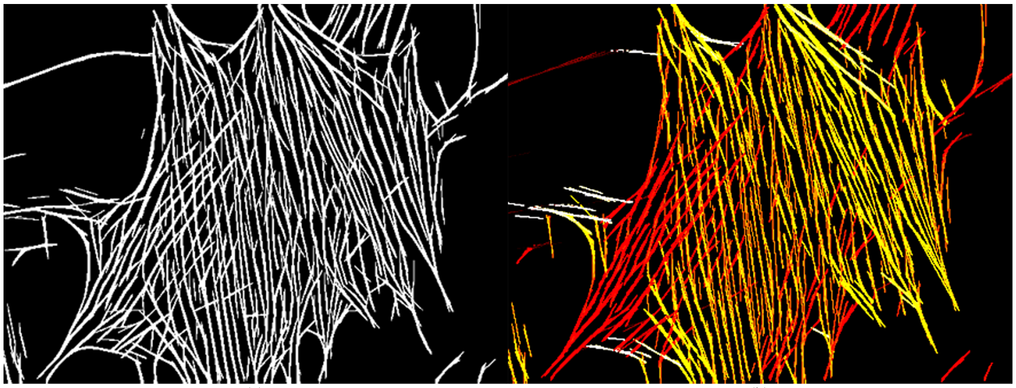

(a) (b)

Figure S7. Extracted filaments with $T_\theta = 2$. (a) Binary image. (b) Individual filaments. The different colors depict different orientations.
